# Influence of Small Changes in Intermolecular Distances on Results of Interaction Energy Decomposition for Stacking Interactions, Hydrogen Bonds, and Antiparallel Interactions

**DOI:** 10.3390/ijms27219436

**Published:** 2026-10-23

**Authors:** Jelena P. Blagojević Filipović, Snežana D. Zarić

**Affiliations:** 1 Innovative Center of the Faculty of Chemistry, University of Belgrade, Studentski trg 12–16, 11000 Belgrade, Serbia; jbfilipovic@chem.bg.ac.rs; 2 Faculty of Chemistry, University of Belgrade, Studentski trg 12–16, 11000 Belgrade, Serbia

**Keywords:** SAPT energy decomposition analysis, stacking interactions, OH/O interactions, CH/O interactions, OH/π interactions, antiparallel OH/OH interactions

## Abstract

A SAPT (Symmetry-Adapted Perturbation Theory) decomposition analysis was performed for various types of noncovalent interactions (stacking, hydrogen bonds, and antiparallel) for various distances of interacting fragments. Although the total interaction energy does not significantly change within the small range around the optimal distance, the energy contributions can significantly change in some systems. This is particularly pronounced for stacking interactions, while for hydrogen bonds and antiparallel interactions the energy contributions do not significantly change around the optimal distance. Thus, the nature of interactions should be carefully interpreted, as small alterations in distances between the interacting fragments can sometimes lead to significant changes in energy contributions, especially for stacking interactions.

## 1. Introduction

The energy decomposition analysis provides insight into the interaction beyond just the total interaction energy itself, since it quantitatively reflects the nature of interactions. The information about the particular energy contributions can be useful for fundamental or practical purposes [1,2,3,4,5,6,7,8,9,10,11,12,13,14,15,16]. Stacked benzene/benzene and cyclohexane/cyclohexane dimers have similar interaction energies (−2.73 kcal/mol [17] and −2.62 kcal/mol [18], respectively). Symmetry-Adapted Perturbation Theory (SAPT) decomposition showed that both benzene/benzene and cyclohexane/cyclohexane dimers have a dominant dispersion contribution [19]. The electrostatic term plays a significant role in both homodimer interactions, but to a slightly larger extent in the cyclohexane dimer. The induction term is quite small in both systems [19]. The heterodimer cyclohexane/benzene system is slightly more stable (−3.27 kcal/mol [20]) due to the more pronounced electrostatic term as a consequence of CH/π interactions, while dispersion is also a dominant energy contribution as in homodimer interactions [19]. Benzene/benzene interaction retains a lot of its maximum strength upon shifting to a large horizontal displacement (around 70% of the maximum strength, when shifted to 5.0 Å; 1 Å equals 10^−10^ m), while cyclohexane/benzene interaction preserves around 40% of its maximum strength at the same offset value. Since dispersion and exchange-repulsion are both short-ranged interactions and they decrease simultaneously with increasing distance of the interacting monomers while having opposite signs, it is sometimes convenient to express their sum (net dispersion), for example when comparing energy decompositions to the one in benzene/benzene stacking [21,22]. The electrostatic term is more dominant than net dispersion in the case of the benzene/benzene stacked dimer, in the most stable geometry, as well as at large offsets. On the other hand, the electrostatic term is also more dominant than the net dispersion term at the most stable geometry of the cyclohexane/benzene dimer, but net dispersion slightly dominates at large offsets [23]. If the electrostatic term is further separated into a multipole expansion term and the electrostatic penetration term, it can be shown that the latter one is dominant [24]. The tendency of benzene/benzene interactions to preserve a larger percentage of their strength at large offsets, compared to benzene/cyclohexane, is reflected in the crystal structure arrangements. The offset distributions of parallel benzene/benzene contacts show a peak at large offsets, but not at smaller offsets [25]. Large offsets are favoured by the possibility of forming additional interactions, as well as significant benzene/benzene interaction strength, which is preserved [23]. On the other hand, phenyl/cyclohexyl contacts show both peaks in smaller and larger offset values [20]. Effects of crystal packing also favour large offsets, but the interaction energy effect supports smaller offsets, since a smaller portion of the interaction energy is preserved at large offsets [23].

Heterocyclic aromatic molecules form stronger stacking interactions than benzene molecules due to the enhanced electrostatic contribution, as a result of the molecule polarization occurring in the presence of electronegative heteroatoms [26]. For example, comparing benzene/benzene, benzene/pyridine and pyridine/pyridine dimers, it can be seen that the pyridine/pyridine dimer is the most stable (−3.99 kcal/mol [27]), since antiparallel interactions are present, followed by the benzene/pyridine (−3.44 kcal/mol [26]) and benzene/benzene dimer (−2.73 kcal/mol [17]). The dispersion term is the major contribution to the total interaction energy in pyridine/pyridine and benzene/pyridine systems, like in the benzene/benzene dimer, as mentioned above. Induction, dispersion and exchange-repulsion terms are reduced in both pyridine/pyridine and benzene/pyridine dimers, compared to the benzene dimer, since the electronegative heteroatom contracts the electron density. As a result of such contraction, the polarizability decreases, as does the repulsion between two interacting molecules [21,22]. The net dispersion term (the sum of dispersion and exchange-repulsion [22]) is attractive in all systems. It is the smallest in benzene/benzene and the largest in the pyridine/pyridine system, which means that the exchange-repulsion contribution is becoming more reduced than the dispersion term [22]. Pyridine/pyridine and benzene/pyridine stacking interactions preserve a significant portion of their maximum strength when shifted to the large offset value (4.5 Å), 53% and 59%, respectively [28]. The large offset values are dominant in the crystal structures of parallel pyridine/pyridine contacts [28].

SAPT energy decomposition analysis of OH/O interactions performed on the optimized water dimer [29] showed that the electrostatic term is dominant (about twice as large as the total interaction energy). Regarding the sum of attractive terms in the total interaction energy, the electrostatic contribution forms more than 60%, while dispersion and induction terms are also important (around 20% and 10% of the attractive part of the interaction energy, respectively). The contribution of the electrostatic term in the attractive part of the interaction energy increases with increasing O∙∙∙H distance, while the induction term decreases at the same time. The exchange-repulsion term is comparable to the electrostatic term, but of opposite sign.

A SAPT energy decomposition of CH/O interactions studied on the methane/water dimer reveals that dispersion is the most dominant attractive contribution to the total interaction energy, followed by the electrostatic and induction components. The exchange-repulsion term exceeds all the attractive terms [30]. The most stable geometry of both OH/O and CH/O interactions is linear, but the CH/O interaction is less sensitive to bending. Although the CH/O interaction is weaker than OH/O (comparing the most stable geometries), the interaction energy decreases more gradually with increasing monomer distance in the case of the CH/O interaction, so the strengths of the OH/O and CH/O interactions can be comparable at particular positions [31]. CH/O interactions are often considered weak, but they can be much stronger when the CH group is a part of a coordinated species [32,33].

Dispersion is the major contribution to attraction in OH/π interactions. Electrostatic interaction is not dominant, but it determines the preferred geometries. The most stable geometry of a benzene/water dimer is a monodentate one, with a hydrogen atom located above the ring centre [34,35]. Exchange-repulsion energy is also quite important in describing conformations and interaction energies of OH/π interactions [36]. On the other hand, a recent study on OH/π and NH/π interactions in the second coordination sphere of the metal complexes [37] emphasizes the importance of the electrostatic contribution in these interaction types, followed by induction and dispersion as the lowest attractive contributions. The energy contributions [37] and total interaction energies [37,38,39] are similar for OH/π and NH/π interactions of the coordinated water and ammonia molecules.

There is a group of attractive interactions between OH-containing species that do not meet the geometric criteria for hydrogen bonds. Thus, analyzing the attractive water/water interactions in the CSD, it is observed that most of them are classical hydrogen bonds, but besides them there are others, most of which have antiparallelly oriented OH bonds. The geometric criteria defined for these interactions reveal 1282 attractive antiparallel OH/OH contacts out of a total of 6729 attractive water/water contacts [40]. The potential energy surface is systematically calculated [41]. The most stable antiparallel OH/OH contacts on the potential energy surface have an interaction energy of −4.2 kcal/mol, while a significant portion of the surface corresponds to interaction energies more negative than −2.0 kcal/mol, meaning that these interactions can be stronger than weak hydrogen bonds. These antiparallel water/water interactions are shown to be a low-barrier transition state between two classical hydrogen bonds with reversed donor–acceptor roles [41]. These interactions can be described as an attraction between two antiparallelly oriented OH dipoles. The antiparallel OH/OH contacts also exist for alcohol/alcohol and alcohol/water interactions, as shown by the CSD search and quantum chemical calculations [42]. The OH group also forms parallel-alignment interactions with aromatic rings [43,44]. The SAPT energy decomposition analysis shows that the most dominant attractive component is electrostatic (ca. 66% of all attractive contributions), dispersion is less dominant (ca. 23% of attractive contributions), while induction is the least dominant attractive contribution (ca. 12%). Electrostatic and dispersion contributions are slightly more pronounced in antiparallel OH/OH interactions than in classical hydrogen bonds (ca. 62% and 20%, respectively), while induction is slightly less pronounced than in classical hydrogen bonds (ca. 18%) [41].

In this work, we performed SAPT analysis of various stacking interactions, various conventional and nonconventional hydrogen bonds (OH/O, CH/O, OH/π), and antiparallel OH/OH interaction with the aim of showing dependence of the SAPT energy components on small changes in distance between interacting monomers. To the best of our knowledge, these are the first results indicating a change in the dominant energy component with a small change in the distance.

## 2. Results and Discussion

Energy decomposition analysis of various stacking interactions (benzene/benzene, benzene/pyridine, pyridine/pyridine and benzene/cyclohexane), various hydrogen bonds (CH/O (methane/water), OH/O (water/water), OH/π (water/benzene), and antiparallel OH/OH (water/water) using the Symmetry-Adapted Perturbation Theory (SAPT) [45] approach was performed. Interaction energies and their components were calculated at optimal intermolecular distances and at somewhat shorter and larger distances.

### 2.1. Stacking Interactions

**Figure 1:**
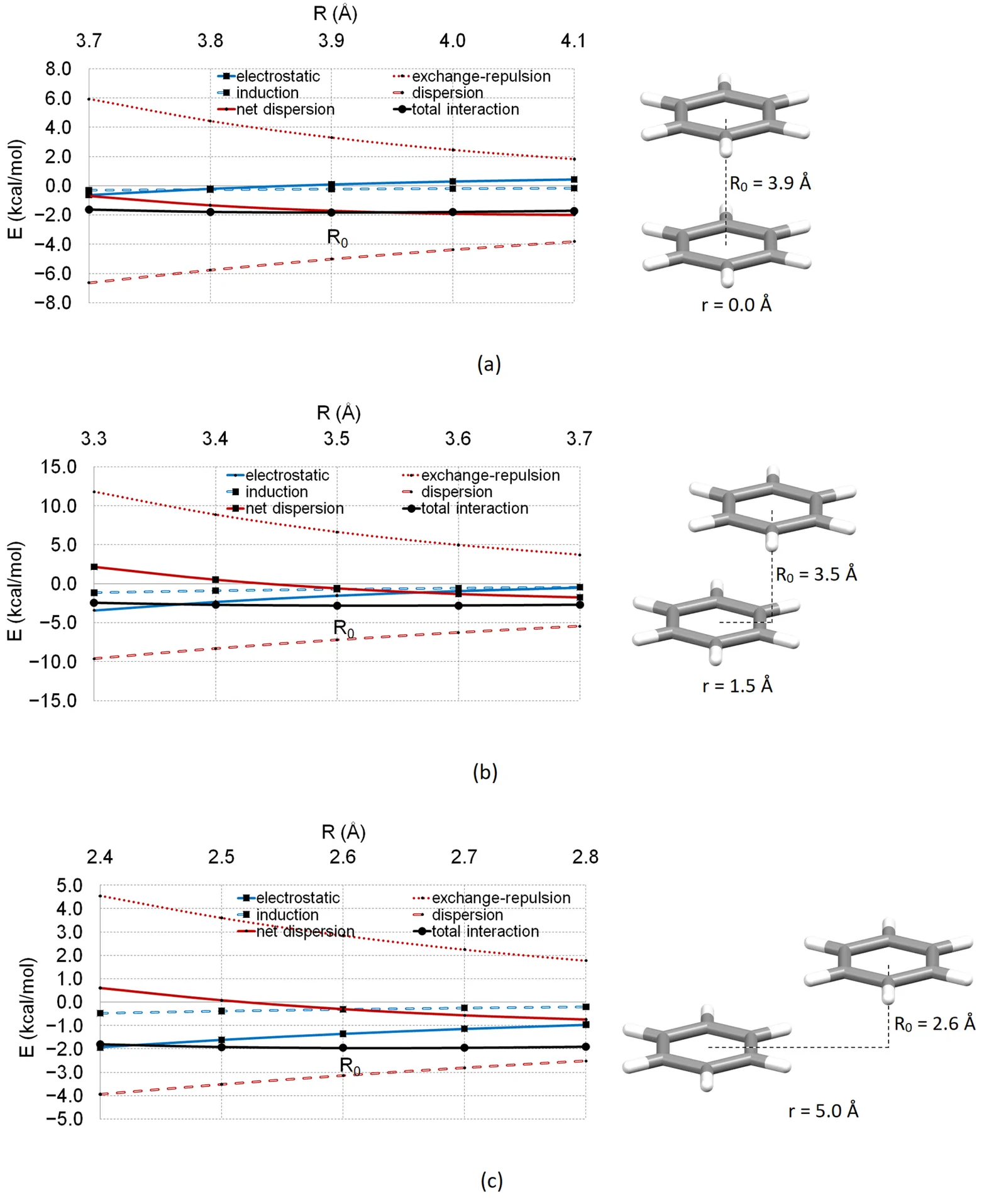
Dependence of the total interaction energies and their components on vertical displacements (*R*) with the corresponding geometries, for benzene/benzene stacking interactions at offset values (**a**) 0.0 Å; (**b**) 1.5 Å; (**c**) 5.0 Å; *r* is a chosen horizontal displacement; *R*_0_ denotes optimal vertical displacement.

**Figure 2:**
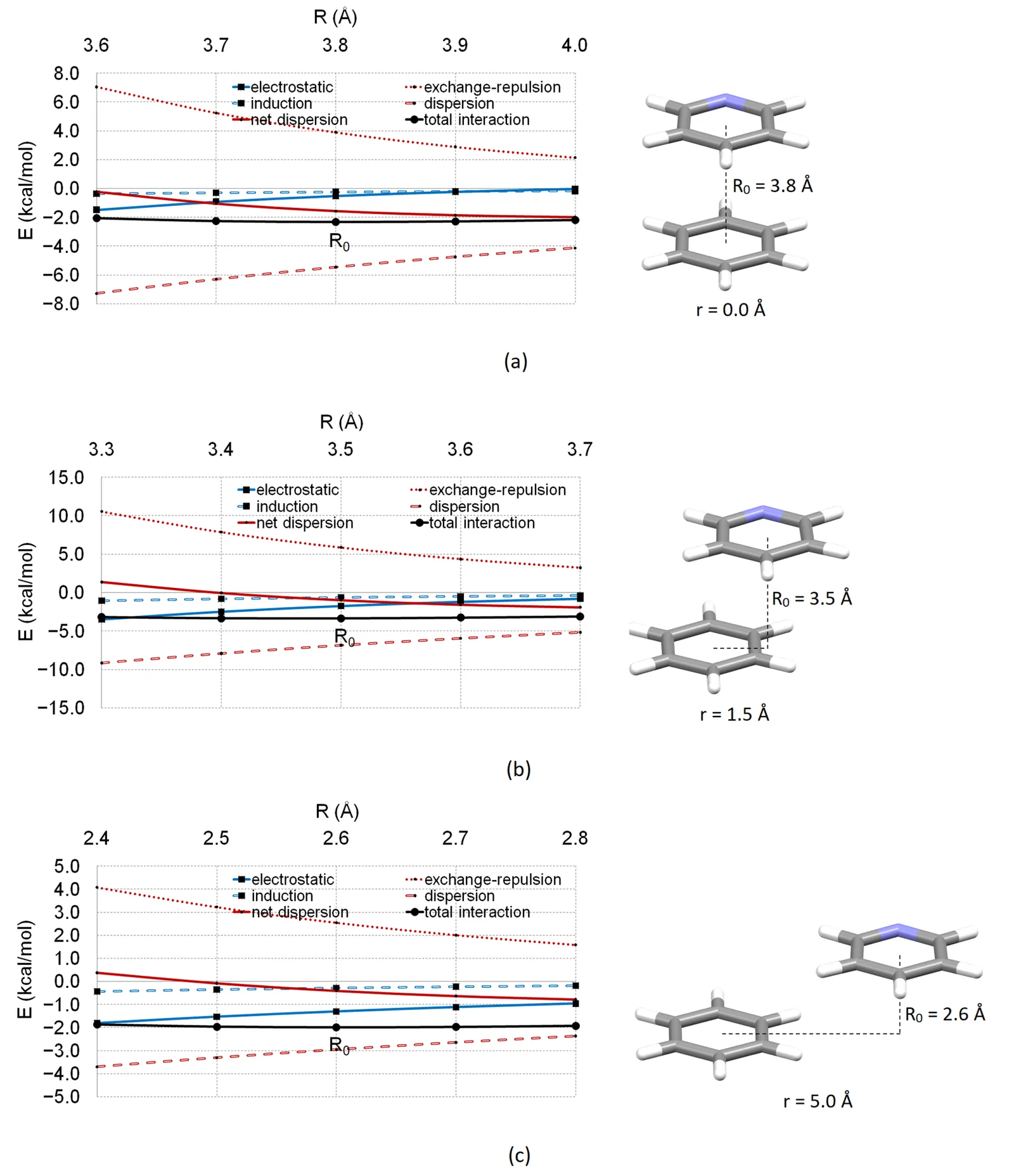
Dependence of the total interaction energies and their components on vertical displacements (*R*) with the corresponding geometries, for benzene/pyridine stacking interactions at offset values (**a**) 0.0 Å; (**b**) 1.5 Å; (**c**) 5.0 Å; *r* is a chosen horizontal displacement; *R*_0_ denotes optimal vertical displacement.

**Figure 3:**
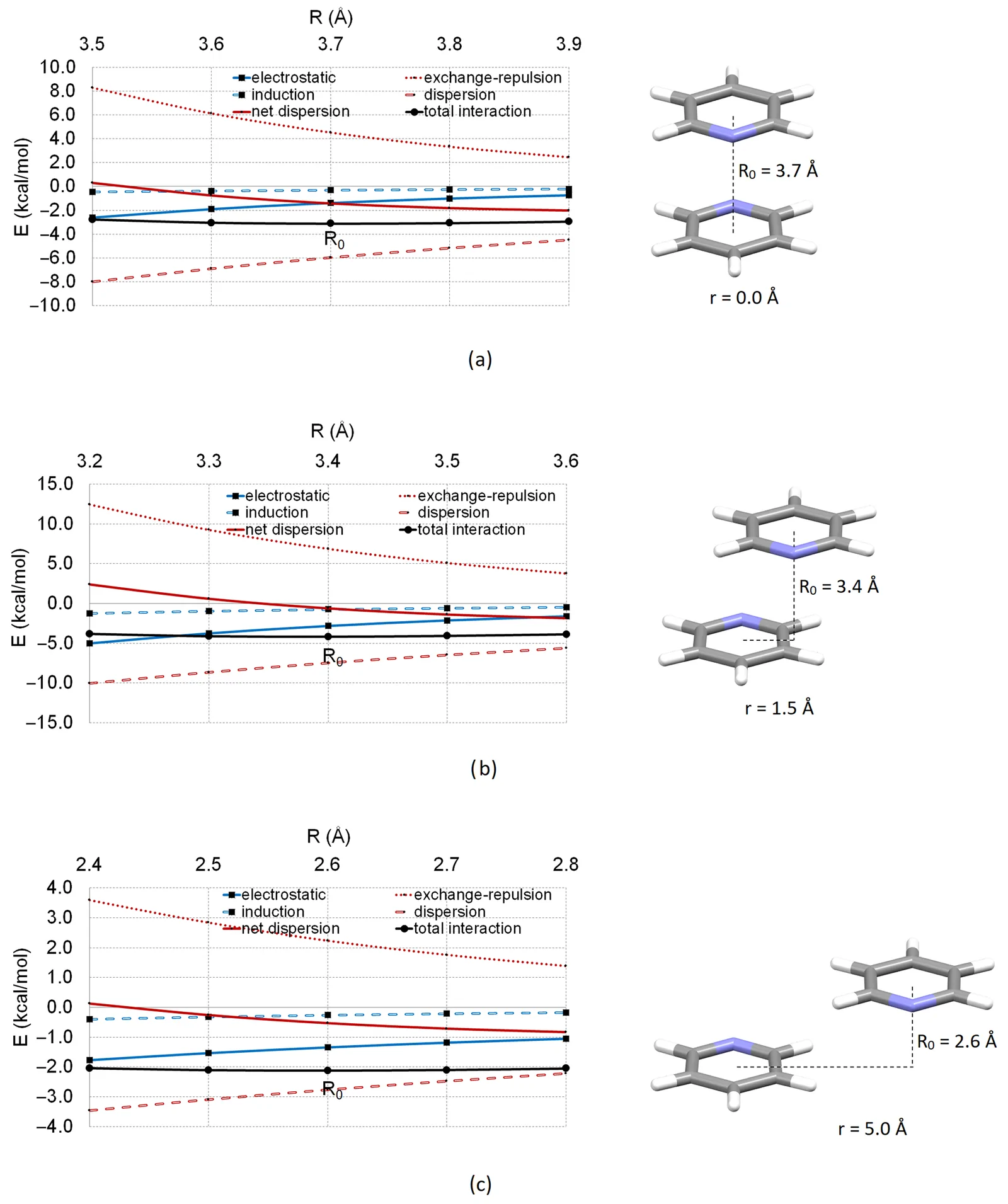
Dependence of the total interaction energies and their components on vertical displacements (*R*) with the corresponding geometries, for antiparallel pyridine/pyridine stacking interactions at offset values (**a**) 0.0 Å; (**b**) 1.5 Å; (**c**) 5.0 Å; *r* is a chosen horizontal displacement; *R*_0_ denotes optimal vertical displacement.

**Figure 4:**
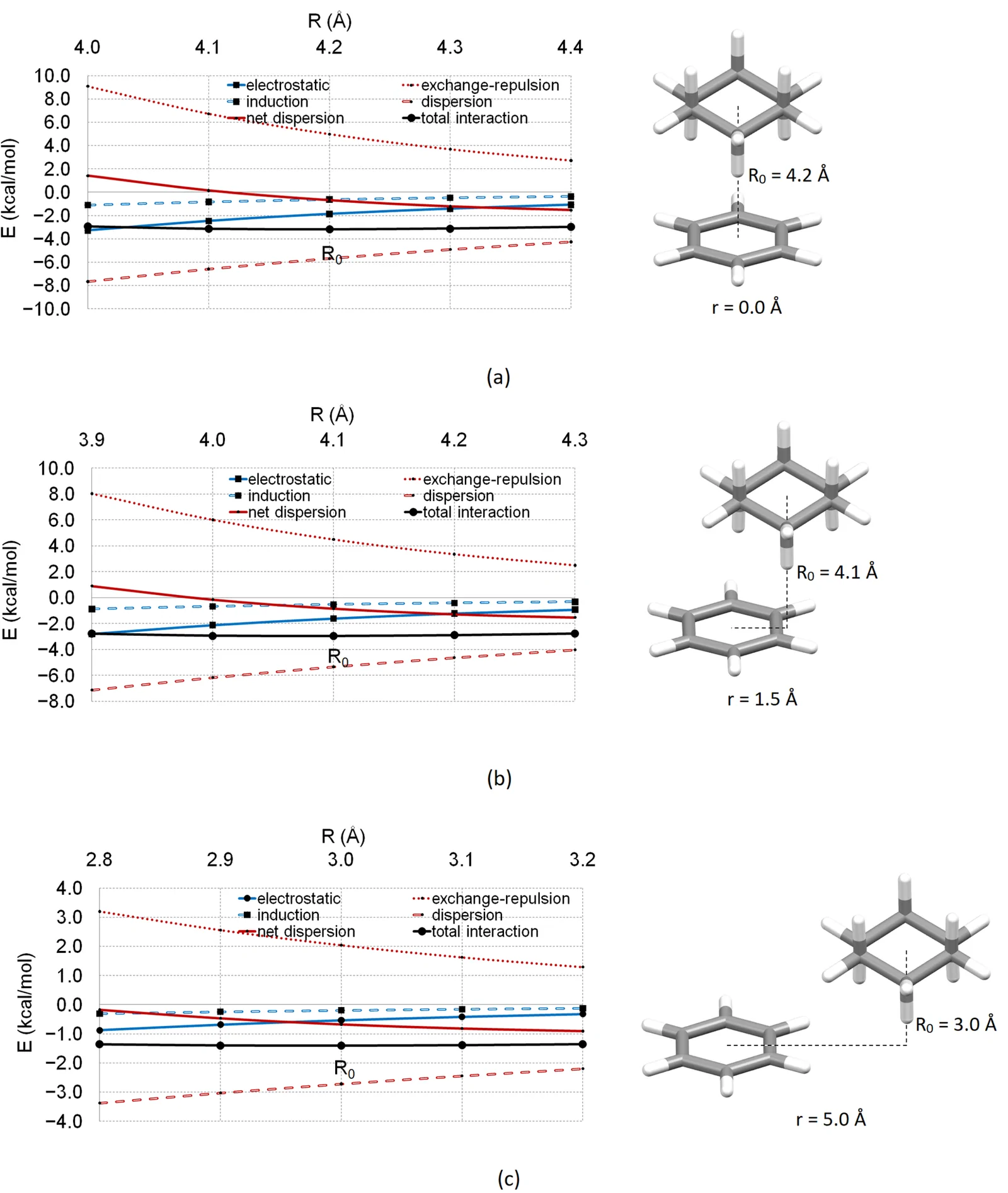
Dependencies of the total interaction energies and their components on vertical displacements (*R*) with the corresponding geometries, for benzene/cyclohexane stacking interactions at offset values (**a**) 0.0 Å; (**b**) 1.5 Å; (**c**) 5.0 Å; *r* is a chosen horizontal displacement; *R*_0_ denotes optimal vertical displacement.

Interaction energies and their components were calculated at optimal vertical displacements and at slightly shorter and larger vertical displacements. The results are shown as potential energy curves representing total SAPT interaction energies and their components depending on vertical displacement (*R*, Figure 1, Figure 2, Figure 3 and Figure 4). Numerical tables of the SAPT energy components are given in the Supplementary Information Section SI-I, while the Cartesian coordinates of the corresponding geometries are given in Section SI-II. Optimal vertical displacement (*R*_0_) corresponds to the energy minimum at a given horizontal displacement value (*r*; Figure 1, Figure 2, Figure 3 and Figure 4).

Total interaction energies practically do not change within the examined range of vertical displacements (Figure 1, Figure 2, Figure 3 and Figure 4), but interaction energy components and, consequently, the nature of the interactions, significantly change. All components that are part of the total SAPT energy (electrostatic, exchange-repulsion, induction and dispersion) weaken with increasing vertical displacements in all stacked systems, except the net dispersion component, which is not a “pure” component but is composed of dispersion and exchange-repulsion. Dispersion becomes less favourable, and the exchange-repulsion term becomes more favourable (due to its positive sign), leading to a more favourable net dispersion, since dispersion drops off to a smaller extent. It should be highlighted that the intersection occurs between net dispersion and other energy components (electrostatic and induction), while the “pure” dispersion term is always dominant across the entire range of the observed distances. Changes in the electrostatic term are more significant than changes in the induction term, which are relatively small in all systems.

When analyzing optimal geometries, dispersion components are dominant in all stacking model systems, as mentioned above (Figure 1, Figure 2, Figure 3 and Figure 4). On the other hand, comparing net dispersion to the electrostatic component, we can see differences between aromatic/aromatic (benzene/benzene, benzene/pyridine and pyridine/pyridine and aromatic/aliphatic (benzene/cyclohexane) interactions. In all aromatic/aromatic interactions, net dispersion interaction is more dominant than electrostatic in sandwich geometries, while the electrostatic interaction is more dominant in displaced geometries (both smaller and larger displacements). The only exception is the pyridine/pyridine sandwich geometry, where both components are similar, since the electrostatic component is enhanced due to the antiparallel dipole arrangement [22]. In aromatic/aliphatic interactions, however, we observe the opposite situation. The electrostatic component dominates in the sandwich geometry [23], and since it originates from CH/π interactions, it significantly decreases in displaced geometries, so that net dispersion even exceeds the electrostatic term at large displacement.

**Benzene/benzene.** In the benzene/benzene sandwich geometry, the only intersection of the curves occurs between the electrostatic and induction components, but that does not significantly change the nature of the interaction since both components are weak (−0.6 kcal/mol and −0.3 kcal/mol at a vertical displacement of 3.7 Å). The intersection occurs because the electrostatic term decreases more rapidly than the induction in this system and even becomes slightly repulsive (0.4 kcal/mol at 4.1 Å) (Figure 1a).

In the parallel-displaced benzene/benzene system, the intersection occurs very close to the calculated optimal vertical displacement (Figure 1b), which significantly changes the nature of the interaction in the proximity of *R*_0_. Namely, the electrostatic term is more dominant than net dispersion at slightly smaller *R* (−2.3 kcal/mol vs. 0.5 kcal/mol at *R* = 3.4 Å), but net dispersion becomes significantly dominant at slightly larger *R* (−0.5 kcal/mol vs. 1.7 kcal/mol at *R* = 3.7 Å). The electrostatic component decreases far more rapidly than the induction component. This effect is even more pronounced than in the sandwich geometry (Figure 1b), since for the parallel-displaced geometry the electrostatic component is three times stronger than the induction component at 3.3 Å (−3.4 kcal/mol vs. −1.2 kcal/mol), while these components are almost equal at *R* = 3.7 Å.

At large horizontal displacement (5.0 Å, Figure 1c), the net dispersion curve intersects the induction curve near *R*_0_ but does not intersect the electrostatic curve. The electrostatic component is significantly more dominant than net dispersion and induction in this system. Namely, the net dispersion value is in the range between 0.6 and −0.7 kcal/mol, induction is even less pronounced (between −0.5 and −0.2 kcal/mol), while the electrostatic term reaches −1.9 kcal/mol at an *R* value of 2.4 Å and decreases to −1.0 kcal/mol at 2.8 Å. The electrostatic contribution decreases more slowly than in the parallel-displaced system, since the nature of electrostatic attraction is not the same. The interaction in the case of parallel-displaced geometry is a quadrupole/quadrupole interaction, while in the case of large horizontal displacement it is the interaction of local dipoles near the aromatic ring edges. Although the curves do not intersect, the electrostatic/net dispersion ratio significantly changes around the *R*_0_ value. Namely, at *R* slightly smaller than optimal (2.4 Å), the electrostatic term is far more dominant than net dispersion, which is even repulsive (−1.9 vs. 0.6 kcal/mol), but they are almost comparable at *R* slightly larger than optimal (2.8 Å, −1.0 vs. −0.7 kcal/mol). The calculated *R*_0_ values for the three studied parallel benzene/benzene geometries are in agreement with the corresponding values found in crystal structures, where the distances between the planes of the interacting benzene rings are dominantly between 3.0 and 4.0 Å for sandwich and parallel-displaced geometries, while dropping to smaller values (2.0–3.0 Å) for parallel large offset geometries [25].

**Benzene/pyridine.** In the sandwich orientation of the benzene/pyridine dimer, the interaction energy has a dominant net dispersion component at the optimal vertical displacement, but the electrostatic/net dispersion ratio dramatically changes in the proximity of *R*_0_ (Figure 2a). Namely, the electrostatic and net dispersion curves intersect at an *R* value around 3.7 Å (0.1 Å shorter than *R*_0_). The net dispersion component is around three times more dominant than the electrostatic component at *R*_0_ (3.8 Å, −1.6 vs. −0.5 kcal/mol), but they are nearly equal at 3.7 Å (−1.0 vs. −0.9 kcal/mol). At 3.6 Å, the electrostatic component significantly exceeds the net dispersion (−1.5 vs. 0.2 kcal/mol). Due to such significant changes in the component ratio while the vertical displacement value changes little, it is very important to carefully determine the exact value of vertical displacement when performing energy decomposition analysis.

In the case of parallel-displaced benzene/pyridine stacking, some effects are similar to those in sandwich geometry. The intersection of net dispersion and electrostatic curves occurs at a slightly larger vertical displacement than optimal (Figure 2b). At the calculated optimal displacement (3.5 Å), the electrostatic component exceeds the net dispersion (−1.7 vs. −1.0 kcal/mol), but at a slightly larger value (3.6 Å), the net dispersion exceeds the electrostatic component (−1.6 vs. −1.2 kcal/mol). At a slightly smaller *R* value (3.4 Å), the electrostatic component is very dominant (−2.5 kcal/mol), while net dispersion drops to zero. The net dispersion curve also intersects the induction curve at an *R* value slightly smaller than optimal. At the *R*0 value (3.5 Å), net dispersion is slightly more favourable than induction (−1.0 vs. −0.6 kcal/mol), but at a slightly shorter *R* value (3.4 Å), induction is more favourable (−0.8 kcal/mol), while net dispersion is negligible. At a slightly larger *R* value (3.6 Å), net dispersion significantly exceeds the induction component (−1.6 vs. −0.5 kcal/mol). Since the component ratio, or even the order of components according to their contribution, significantly changes in a small range of vertical displacements (3.5 ± 0.1 Å), it is crucial to precisely determine the vertical displacement value.

At a large horizontal displacement in the case of benzene/pyridine stacking (Figure 2c), the net dispersion and induction curves intersect near the optimal vertical displacement value, but their contribution is quite small, since it does not exceed −0.6 kcal/mol in the range ±0.1 Å around *R*_0_. Similar to benzene/benzene stacking, in benzene/pyridine stacking the electrostatic component drops off more rapidly in case of sandwich or parallel-displaced geometries than in case of large offset geometries. Thus, the intersection of electrostatic and net dispersion curves does not occur. Namely, the electrostatic component in benzene/pyridine sandwich geometry (Figure 2a) drops off from −1.5 kcal/mol to 0.0 kcal/mol within the *R* value range ±0.2 Å around the optimal value. The electrostatic component is even more attractive for the parallel-displaced geometry of the benzene/pyridine stacking (−1.7 kcal/mol at *R*_0_, Figure 2b) and drops off from −3.5 kcal/mol to −0.8 kcal/mol within the *R* value range ±0.2 Å around the optimal value, which is more than four times lower. Large offset geometry is governed by local C-H dipoles at the ring edges and has the weakest total energy of −2.0 kcal/mol among the three observed geometries. The electrostatic component *R*_0_ is −1.3 kcal/mol and drops off from −1.8 to −1.0 kcal/mol, which is a more gradual change. Similar to the benzene/benzene system, the electrostatic/net dispersion ratio changes, although the intersection does not occur.

**Pyridine/pyridine.** In the case of antiparallel sandwich geometry, the net dispersion curve intersects the electrostatic curve at the optimal vertical displacement (3.7 Å, Figure 3a); thus, similarly to the benzene/pyridine sandwich geometry, interaction energy contributions substantially change within the small range of vertical displacements around the optimal value. The electrostatic component (−1.9 kcal/mol) is more attractive than net dispersion (−0.8 kcal/mol) at slightly smaller *R* (3.6 Å), but net dispersion is more dominant than the electrostatic component at slightly larger *R* (3.8 Å, −1.8 vs. −1.0 kcal/mol). The net dispersion curve also intersects the induction curve at a slightly shorter vertical displacement than optimal, but this is of less importance due to the small induction value (−0.4 kcal/mol or less).

In the case of the parallel-displaced *anti* pyridine/pyridine system, unlike the parallel-displaced benzene/benzene and benzene/pyridine systems, where the net dispersion curve intersects the electrostatic curves at vertical displacement values very close to optimal, the net dispersion curve intersects the electrostatic curve at a slightly larger *R* (Figure 3b), so the electrostatic/net dispersion ratio significantly changes in the range of vertical displacements between the optimal (3.4 Å) and slightly larger (3.6 Å) values. At the optimal vertical displacement (3.4 Å), the electrostatic component (−2.8 kcal/mol) is much more dominant than net dispersion (−0.6 kcal/mol), while net dispersion dominates at 3.6 Å (−1.8 kcal/mol vs. −1.6 kcal/mol). The net dispersion curve also intersects the induction curve around *R*_0_, but that is less important due to a small induction contribution (less than −1.2 kcal/mol in the whole range of vertical displacements, Figure 3b).

At large horizontal displacements of antiparallel pyridine/pyridine stacking, the net dispersion curve intersects the induction curve at a vertical displacement of 2.5 Å, which is slightly shorter than optimal (2.6 Å, Figure 3c), but as in previous systems, this is less important, since the induction contribution is nearly constant and quite small (less than −0.4 kcal/mol). Similar to the benzene/benzene and benzene/pyridine systems in the large-offset pyridine/pyridine antiparallel system, the electrostatic contribution drops off more slowly than in sandwich and parallel-displaced systems, so none of the component curves intersect the electrostatic curve. However, the electrostatic/net dispersion ratio significantly changes around the optimal vertical displacement (Figure 3c). The electrostatic component is significant at 2.4 Å (−1.8 kcal/mol), while net dispersion is repulsive (0.1 kcal/mol), but they are comparable at 2.8 Å (−1.0 vs. −0.8 kcal/mol). Similar to benzene/benzene interactions, in the case of pyridine/pyridine parallel interactions, the *R*_0_ values calculated in this work are in the range dominantly observed in the crystal structures, i.e., around 3.5 Å for horizontal displacements close to 0.0 and 1.5 Å and in the range 2.5–3.5 Å for horizontal displacements around 5.0 Å [28].

**Benzene/cyclohexane.** In sandwich geometry (Figure 4a), the net dispersion curve intersects the induction curve nearly at the optimal vertical displacement value (4.2 Å) and the electrostatic curve at a slightly larger value (4.3 Å), which makes a precise determination of optimal vertical displacement very important in SAPT analysis. Thus, at *R*_0_ the electrostatic term (−1.9 kcal/mol) is far more dominant than net dispersion (−0.7 kcal/mol) and the induction term (−0.6 kcal/mol). At a slightly larger *R* value (4.3 Å), the electrostatic and net dispersion terms are similar (−1.4 kcal/mol vs. −1.2 kcal/mol), while induction is much less attractive (−0.5 kcal/mol). At a bit larger *R* (4.4 Å), net dispersion significantly surpasses the electrostatic term (−1.5 vs. −1.1 kcal/mol), while the induction term is even less attractive (−0.4 kcal/mol).

Similar preferences are present in the parallel-displaced system (Figure 4b), since the net dispersion curve intersects the induction curve at the vertical displacement value very close to optimal (4.1 Å) and the electrostatic curve at a slightly larger value (close to 4.2 Å). Thus, energy contributions significantly change around *R*_0_. Net dispersion and induction terms are similar at *R*_0_ (−0.8 and −0.5 kcal/mol, respectively), while the electrostatic term is much more attractive (−1.6 kcal/mol). At a slightly larger *R* value (4.2 Å), the electrostatic and net dispersion terms are similar (−1.2 and −1.3 kcal/mol, respectively), while the induction contribution is much smaller (−0.4 kcal/mol). On the other hand, at 4.3 Å, the net dispersion term (−1.5 kcal/mol) surpasses the electrostatic (−0.9 kcal/mol) and induction term (−0.3 kcal/mol).

At large offset benzene/cyclohexane system (Figure 4c), the net dispersion term is attractive in the whole range of vertical displacement values. The net dispersion curve intersects the induction and electrostatic curves at *R* values smaller than optimal, but the values of these energy terms are quite small in this system (smaller than −1.0 kcal/mol over the whole range of *R* values).

### 2.2. Hydrogen Bonds and Antiparallel Interactions: CH/O, OH/O, OH/π and Antiparallel OH/OH Interactions

**Figure 5:**
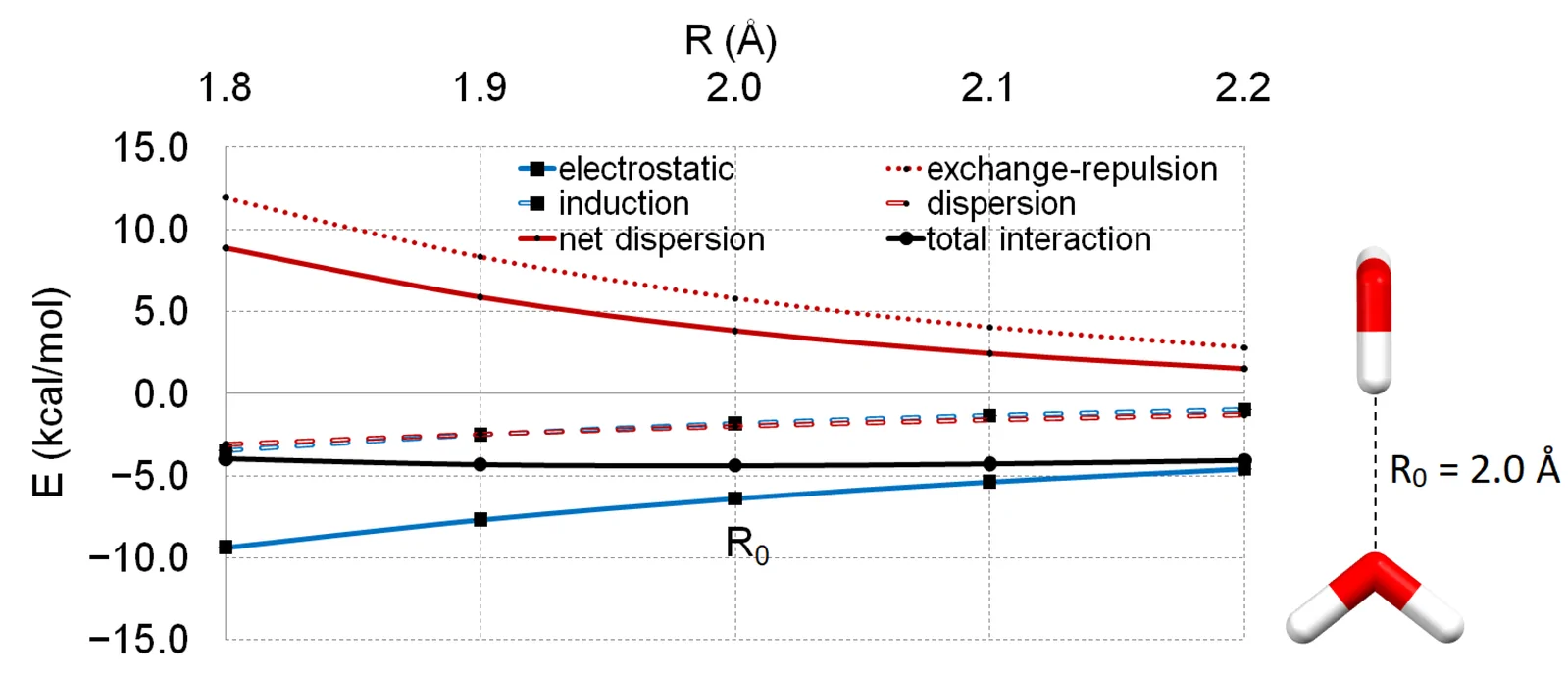
Dependence of the total interaction energies and their components on vertical displacements (*R*) with the corresponding geometries, for water/water OH/O interaction; *R*_0_ denotes optimal vertical displacement.

**Figure 6:**
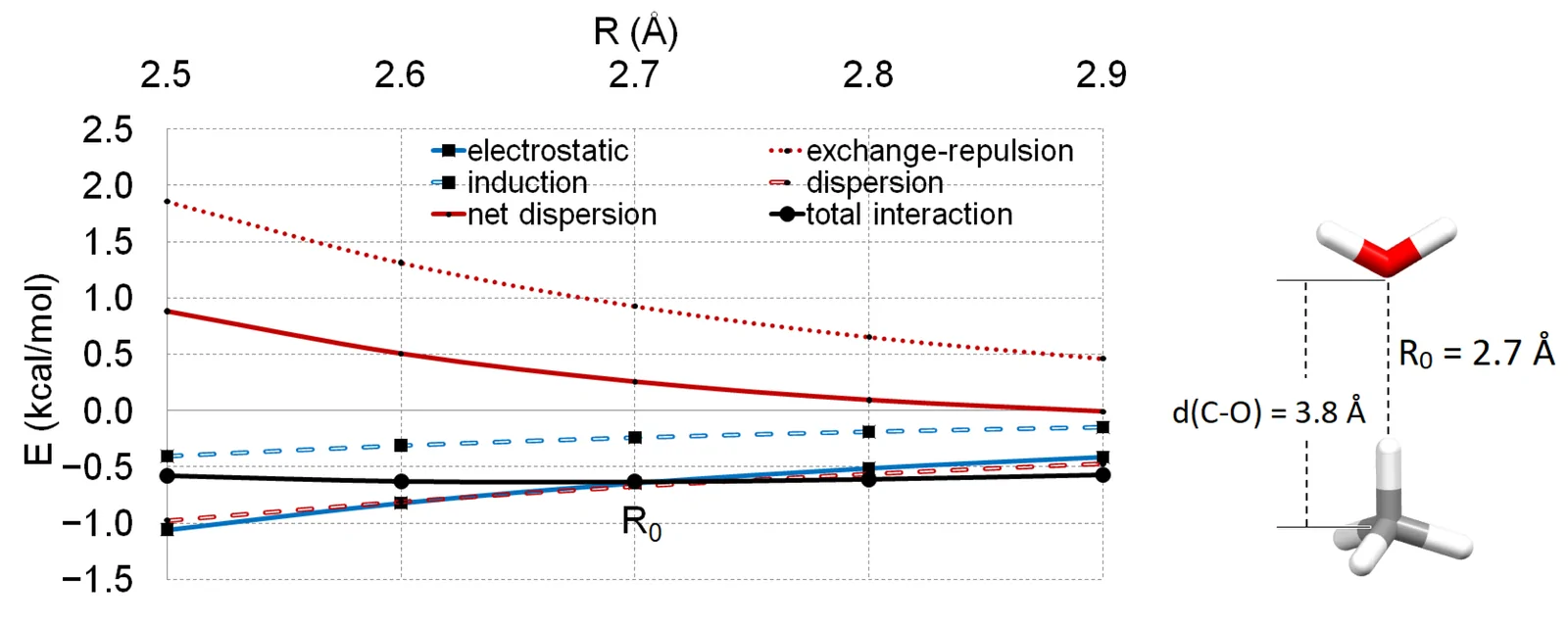
Dependence of the total interaction energies and their components on vertical displacements (*R*) with the corresponding geometries, for methane/water CH/O interaction; *R*_0_ denotes optimal vertical displacement.

**Figure 7:**
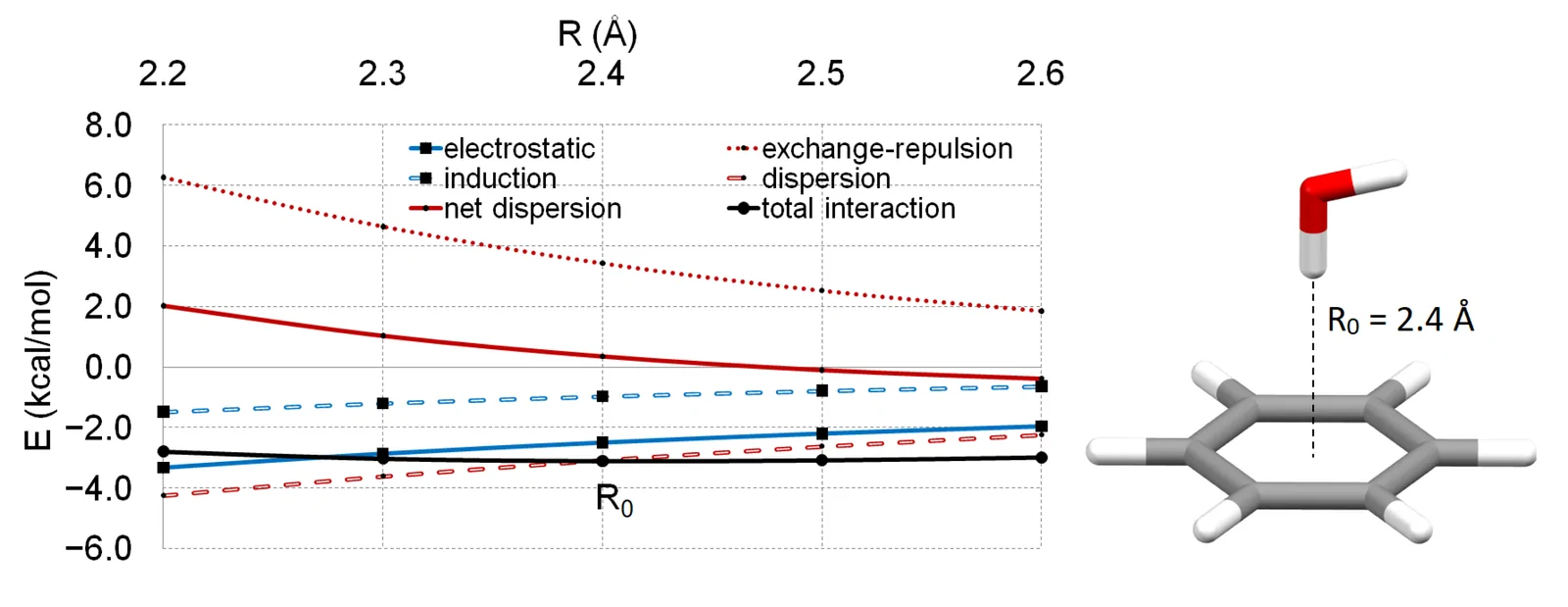
Dependence of the total interaction energies and their components on vertical displacements (*R*) with the corresponding geometries, for water/benzene OH/π interaction; *R*_0_ denotes optimal vertical displacement.

**Figure 8:**
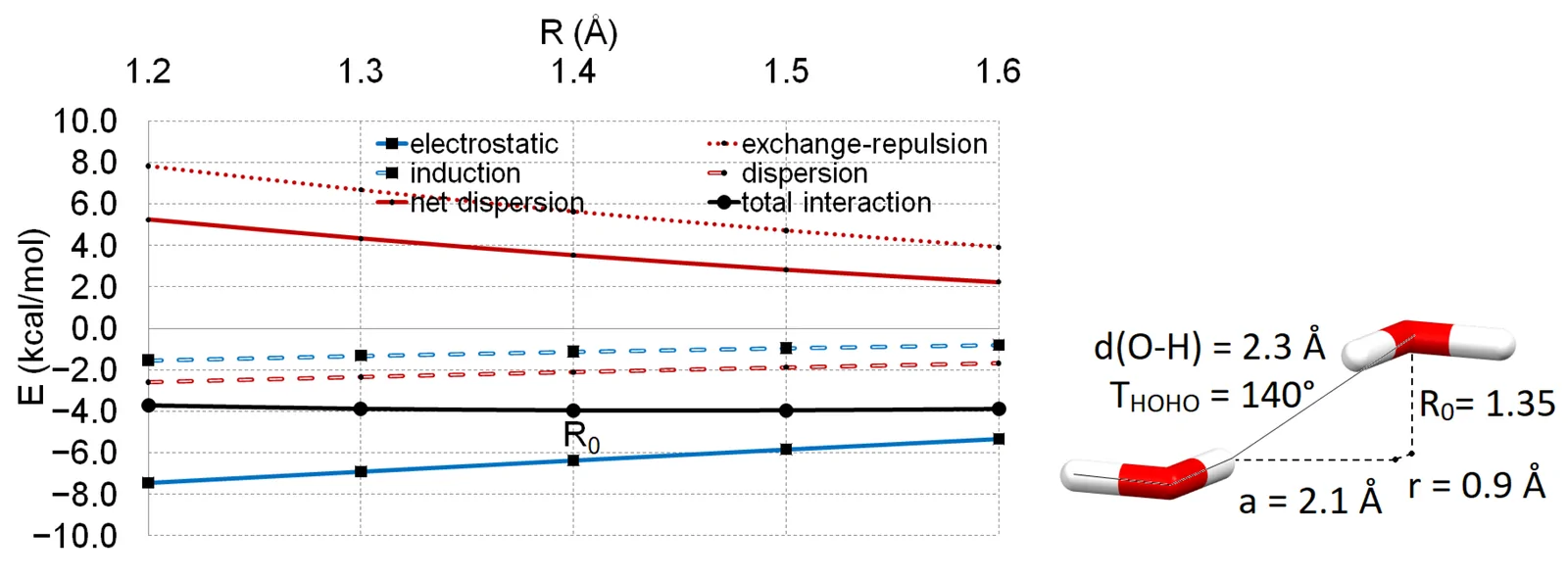
Dependence of the total interaction energies and their components on vertical displacements for the water/water antiparallel OH/OH interaction with the corresponding geometry; the geometry is completely defined by horizontal displacements *a* and *r* and the torsion angle *T*_HOHO_; *R*_0_ denotes optimal vertical displacement.

OH/O, CH/O, OH/π and antiparallel OH/OH interactions have a significantly different nature from stacking interactions, which is reflected in different interaction energy composition. Similar to stacking interactions, total interaction energies remain constant in the observed range of vertical displacements (Figure 5, Figure 6, Figure 7 and Figure 8).

The calculated *R*_0_ values are mostly comparable to the experimentally measured values. Namely, O-H distances in classical hydrogen bonds in water/water contacts found in crystal structures are mostly below 2.0 Å [40], similar to the calculated *R*_0_ value (Figure 5). C-O distances measured in various methane hydrate phases are in the range 3.2–3.7 Å [46], somewhat less than in the most stable system calculated in this work (3.8 Å, Figure 6). Distances between the hydrogen atom and the benzene ring centre are mostly found in the broad range 2.4–3.5 Å [38], while the calculated *R*_0_ value in this work is 2.4 Å (Figure 7). However, O-H distances found in crystal structures of the antiparallel water/water contacts are mostly larger than 3.0 Å [40], which is significantly different than the calculated *R*_0_ value (2.3 Å, Figure 8). This is due to the effects of crystal packing, since the formation of additional interactions is possible in slipped geometries.

With increasing vertical displacements, electrostatic and net dispersion components have opposite tendencies, as in the case of stacking interactions, since the electrostatic component becomes less favourable, while the net dispersion component becomes more favourable. The induction component is small, except for OH/O interactions, which become significantly less attractive with increasing distance between the interacting species. Dispersion becomes less favourable and exchange-repulsion more favourable, as in the case of stacking interactions.

Unlike the stacked systems, the nature of OH/O, CH/O, OH/π and antiparallel OH/OH interactions does not significantly change with changes in vertical displacement. Intersection practically does not occur (Figure 5, Figure 6, Figure 7 and Figure 8), except in OH/O and CH/O systems (Figure 5 and Figure 6), where dispersion and induction curves (OH/O system) and electrostatic and dispersion curves (CH/O system) almost overlap, but that does not lead to significant changes in the nature of interactions.

**OH/O interactions.** In the case of OH/O interactions, the electrostatic component significantly dominates in the whole range of observed vertical displacements. Induction and dispersion curves practically overlap. Net dispersion is always repulsive, since exchange-repulsion is dominant over the dispersion component (Figure 5).

**CH/O interactions.** In the case of CH/O interactions, the electrostatic and dispersion components drop off a bit more rapidly than the induction component. Net dispersion is repulsive in this region and drops to zero with increasing vertical displacement, since dispersion and exchange-repulsion components nearly equalize (Figure 6).

**OH/π.** OH/π interaction is mainly dispersive in nature, with a significant electrostatic contribution, which is nearly as important as dispersion. Net dispersion is mostly repulsive, but becomes attractive at larger vertical displacements, since exchange-repulsion weakens more than dispersion (Figure 7).

**Antiparallel OH/OH.** In the case of antiparallel OH/OH interactions, the electrostatic component is quite dominant in the whole range of vertical displacements, and the electrostatic curve is practically parallel to the dispersion and the induction curves, which means that the contributions of attractive terms do not significantly change. The dispersion component is more dominant than induction in the whole range of vertical displacements. Net dispersion is always repulsive, since dispersion is smaller than the exchange-repulsion contribution (Figure 8).

The physical explanation for the observed differences lies in different contact areas of the interacting fragments. Namely, in stacking interactions, dispersion is quite dominant in all systems (Figure 1, Figure 2, Figure 3 and Figure 4), since it is favoured by larger contact area, in contrast to the electrostatic component, which is not dominant. Therefore, the net dispersion curve will likely intersect the electrostatic curve. On the other hand, a smaller contact area in hydrogen-bonded and antiparallel OH/OH systems does not favour the dispersion component, while the electrostatic component is localized and dominant (OH/O, Figure 5 and antiparallel OH/OH, Figure 8) or comparable (CH/O, Figure 6 and OH/π, Figure 7) to the dispersion. Consequently, the electrostatic curve will significantly exceed the net dispersion curve across the entire distance range.

### 2.3. Optimized Systems

Since the monomer geometries were kept rigid, the interaction energies and their contributions can slightly differ compared to the model, where the atomic positions are relaxed. According to the Interacting Quantum Atoms (IQA) theory, both interatomic and intraatomic energy changes contribute to the total energy of the system and consequently to the interaction energy [47,48,49]. Therefore, the most stable geometries obtained by the SAPT analysis of the rigid systems were fully optimized to estimate the relaxation error. The SAPT energy decomposition is also performed on optimized systems. The Cartesian coordinates of the optimized systems are given in S-II, while the obtained geometries are shown in the SI-III, Figures SIII-1–SIII-8. The SAPT interaction energy decompositions calculated on the optimized systems are compared to the SAPT decompositions of the curve minima (Tables SIII-1–SIII-8).

In some systems, the optimized geometries are significantly different than the starting ones, including benzene/benzene and benzene/cyclohexane systems at large horizontal displacements, all benzene/pyridine and pyridine/pyridine systems, and benzene/water OH/π systems. These rigid geometries used for SAPT analysis are not close to local minima on the potential surface; however, these geometries are important since they exist in crystal structures quite frequently, as was shown in previous studies [20,25,27]. In the case of the large-offset benzene/benzene system, the optimized geometry is the same as the optimized parallel-displaced (r = 1.5 Å) benzene/benzene, which is close to the starting benzene/benzene parallel-displaced geometry (Figure SIII-1). In the second group of systems, the optimized geometries are similar to the starting ones, but the interaction energies are not similar. This is the case in the small-offset benzene/benzene (0.0 and 1.5 Å), benzene/cyclohexane systems (0.0 Å), mostly since the ring planes are not ideally parallel in the optimized dimers, and water/water OH/O, where the hydrogen bond length is slightly changed (Figure SIII-5).

The most interesting systems are those where the interaction energies remain similar after the optimization. This is the case in benzene/cyclohexane parallel-displaced (r = 1.5 Å) stacking, methane/water CH/O and water/water antiparallel OH/OH interaction. The interaction energy differences between the optimized and starting systems are only 0.15, 0.14 and 0.04 kcal/mol, respectively, but the energy contributions show much more significant changes (Tables SIII-4, SIII-6 and SIII-8), similarly to the conclusions obtained by the potential energy curve analysis (Figure 1, Figure 2, Figure 3, Figure 4, Figure 5, Figure 6, Figure 7 and Figure 8).

## 3. Methodology

The Symmetry-Adapted Perturbation Theory (SAPT2 + 3) [45,50,51] interaction energies were calculated with the def2-TZVPPD basis set [52] using the PSI4 program package [53] on model systems representing different interaction types. Model systems for stacking interactions were parallel molecules, with various horizontal and vertical displacements. CH/O, OH/O and OH/π interactions are studied on model systems of methane/water, water/water and water/benzene dimers by varying vertical displacements between the interacting molecules. The model system for antiparallel OH/OH interactions is the most stable calculated water/water dimer formed by antiparallel OH/OH geometry [41], which is defined by parallel molecular planes, a torsion angle *T* of 140°and *a* and *r* displacements of 2.1 and 0.9 Å, respectively (Figure 8). Consequently, the optimal vertical displacement (*R*_0_, as defined in this paper) is 1.35 Å. The horizontal displacements (*a* and *r*) are held constant, and only the vertical displacement of the parallel molecule planes (*R*) is varied (torsion angle *T* consequently varies). Monomer geometries in all systems are optimized using Møller–Plesset perturbation theory of the second order (MP2 [54]) with the cc-pVTZ basis set [55]. Geometries of the interacting monomers were rigid in the potential energy curve calculations, and only their mutual orientations were varied. Optimizations of the potential curve minima geometries were performed using the B3LYP functional [56,57] and adding the D3 version of Grimme dispersion with Becke–Johnson damping [58] at the def2-TZVP level [59].

## 4. Conclusions

SAPT energy decomposition analysis was performed for several representative types of noncovalent interactions, including stacking interactions (benzene/benzene, benzene/pyridine, pyridine/pyridine, and benzene/cyclohexane), hydrogen bonds (OH/O in water/water, CH/O in methane/water, and OH/π in benzene/water), and antiparallel (OH/OH in water/water) interactions. For each system, the total interaction energy and its individual components (electrostatic, exchange-repulsion, induction, and dispersion) were calculated at several intermolecular distances around the optimal distance.

As expected, the total interaction energy changes are very small within the relatively narrow distance range around the optimal distance. In contrast, the individual SAPT components can exhibit substantially larger variations. Consequently, even when the total interaction energy remains relatively stable, the relative contributions of the different components change appreciably. This effect is particularly pronounced for some interaction types and can be visualized by examining the intersections of the component curves.

For this analysis, particular attention was given to the electrostatic and net dispersion contributions, where the latter is defined as the sum of the attractive dispersion and repulsive exchange terms. Both dispersion and exchange-repulsion are short-range contributions, and their combination provides a useful measure of the net dispersive stabilization. An intersection of the electrostatic and net dispersion curves is observed for all investigated stacking systems. This behaviour indicates that relatively small changes in intermolecular distance around the optimal geometry can substantially alter the balance between electrostatic and dispersion contributions. Thus, although the total interaction energy may vary only modestly, the contributions responsible for stabilization change considerably within this short distance range.

The hydrogen-bonded and antiparallel OH/OH–water/water systems exhibit markedly different behaviour. No significant intersections of the SAPT component curves are observed within the investigated distance range. The only exceptions are two cases in which the curves nearly overlap: the electrostatic and dispersion contributions in the CH/O system and the dispersion and induction contributions in the OH/O system.

Overall, these results indicate that the balance between the different interaction components is considerably less sensitive to small changes in intermolecular distance for hydrogen bonds and antiparallel OH/OH–water/water interactions than for stacking interactions. These findings highlight the importance of carefully interpreting the results of the energy decomposition analysis, with reference to the geometry of interacting fragments, particularly for stacked systems.

## Data Availability

The data that support the findings of this study are available within the article and its Supplementary Materials or from the corresponding author upon reasonable request.

## Supplementary Materials

The following supporting information can be downloaded at: https://www.mdpi.com/article/10.3390/ijms27219436/s1.

## References

[1] Pastorczak E., Corminboeuf C. (2017). Found in translation: Quantum chemical tools for grasping non-covalent interactions. J. Chem. Phys..

[2] Das A.K., Liu M., Head-Gordon T. (2022). Development of a many-body force field for aqueous alkali metal and halogen ions: An energy decomposition analysis guided approach. J. Chem. Theory Comput..

[3] Lu Z., Zhou N., Wu Q., Zhang Y. (2011). Directional dependence of hydrogen bonds: A density-based energy decomposition analysis and its implications on force field development. J. Chem. Theory Comput..

[4] McDaniel J.G., Schmidt J.R. (2016). Next-generation force fields from symmetry-adapted perturbation theory. Annu Rev. Phys. Chem..

[5] Deng S., Wang Q., Ren P. (2017). Estimating and modeling charge transfer from the SAPT induction energy. J. Comput. Chem..

[6] Mao Y., Loipersberger M., Horn P.R., Das A., Demerdash O., Levine D.S., Veccham S.P., Head-Gordon T., Head-Gordon M. (2021). From intermolecular interaction energies and observable shifts to component contributions and back again: A tale of variational energy decomposition analysis. Annu Rev. Phys. Chem..

[7] Yang Z., Feng X., Song S., Yang X., Wang Y., Zhang Q. (2026). Binding energy in clusters (BEC): An effective bridge connecting the microscopic structure and macroscopic properties of molecular crystal materials. J. Mater. Chem. A..

[8] Tian J., Li J., Chen H., He P., Zhang Y., Wang P., Wei D., Pi W., Ma J., Sun Y. (2026). Synergistic pore affinity engineering in tobacco stem-derived carbon via magnesium citrate dual activation for high hydrogen storage. Carbon.

[9] Ibrahim M.A.A., Rady A.-S.S.M., Sidhom P.A., Sayed S.R.M., Ibrahim K.E., Awad A.M., Shoeib T., Mohamed L.A. (2024). A comparative DFT investigation of the adsorption of temozolomide anticancer drug over beryllium oxide and boron nitride nanocarriers. ACS Omega.

[10] Tsuzuki S., Ono R., Inoue S., Matsuoka S., Hasegawa T. (2024). Origin of the intermolecular forces that produce donor–acceptor stacks in π-conjugated charge-transfer complexes. Commun. Chem..

[11] Ibrahim M.A.A., Rady A.S.M., Moussa N.A.M., Ahmed M.N., Sidhom P.A., Shawky A.M., Alqahtani A.M., Mohamed L.A. (2023). Investigation of aluminum nitride nanocarrier for drug delivery process of favipiravir: A DFT study. J. Mol. Liq..

[12] Kumawat R.L., Sherrill C.D. (2023). High-order quantum-mechanical analysis of hydrogen bonding in Hachimoji and natural DNA base pairs. J. Chem. Inf. Model..

[13] Hapka M., Krzemińska A., Modrzejewski M., Przybytek M., Pernal K. (2023). Efficient calculation of the dispersion energy for multireference systems with Cholesky decomposition: Application to excited-state interactions. J. Phys. Chem. Lett..

[14] Hoffman M.P., Xantheas S.S. (2025). Competition between Hydrogen and Chalcogen Bonding in Homodimers of Chalcogen Hydrides (H_2_X)_2_, X = O, S, Se, Te. J. Am. Chem. Soc..

[15] Gao Y.-J., Chen W.-K., Fang W.-H. (2024). A green host–guest protocol to improve water solubility of fluorescent dyes. J. Phys. Chem. Lett..

[16] Kříž K., Van der Spoel D. (2024). Quantification of anisotropy in exchange and dispersion interactions: A simple model for physics-based force fields. J. Phys. Chem. Lett..

[17] Lee E.C., Kim D., Jurecka P., Tarakeshwar P., Hobza P., Kim K.S. (2007). Understanding of assembly phenomena by aromatic-aromatic interactions: Benzene dimer and the substituted systems. J. Phys. Chem. A.

[18] Kim K.S., Karthikeyan S., Jiten Singh N. (2011). How different are aromatic π interactions from aliphatic π interactions and non-π stacking interactions?. J. Chem. Theory Comput..

[19] Cabaleiro-Lago E.M., Rodriguez-Otero J. (2017). σ-σ, σ-π, and π-π stacking interactions between six-membered cyclic systems. Dispersion dominates and electrostatics commands. ChemistrySelect.

[20] Ninković D.B., Vojislavljević-Vasilev D.Z., Medaković V.B., Hall M.B., Brothers E.N., Zarić S.D. (2016). Aliphatic-aromatic stacking interactions in cyclohexane–benzene are stronger than aromatic-aromatic interaction in benzene dimer. Phys. Chem. Chem. Phys..

[21] Hohenstein E.G., Sherrill C.D. (2009). Effects of heteroatoms on aromatic π-π interactions: Benzene-pyridine and pyridine dimer. J. Phys. Chem. A.

[22] Sherrill C.D. (2013). Energy component analysis of π interactions. Acc. Chem. Res..

[23] Ninković D.B., Blagojević Filipović J.P., Hall M.B., Brothers E.N., Zarić S.D. (2020). What is special about aromatic—Aromatic interactions? significant attraction at large horizontal displacement. ACS Cent. Sci..

[24] Cabaleiro-Lago E.M., Rodríguez-Otero J., Vázquez S.A. (2022). Electrostatic penetration effects stand at the heart of aromatic π interactions. Phys. Chem. Chem. Phys..

[25] Ninković D.B., Janjić G.V., Veljković D.Ž., Sredojević D.N., Zarić S.D. (2011). What Are the Preferred Horizontal Displacements in Parallel Aromatic–Aromatic Interactions? Significant Interactions at Large Displacements. ChemPhysChem.

[26] Řezáč J., Riley K.E., Hobza P. (2011). S66: A well-balanced database of benchmark interaction energies Relevant to biomolecular structures. J. Chem. Theory Comput..

[27] Ninković D.B., Janjić G.V., Zarić S.D. (2012). Crystallographic and ab initio study of pyridine stacking interactions. Local Nature of hydrogen bond effect in stacking interactions. Cryst. Growth Des..

[28] Ninković D.B., Andrić J.M., Zarić S.D. (2013). Parallel interactions at large horizontal displacement in pyridine-pyridine and benzene-pyridine dimers. ChemPhysChem.

[29] Wang B., Jiang W., Dai X., Gao Y., Wang Z., Zhang R.-Q. (2016). Molecular orbital analysis of the hydrogen bonded water dimer. Sci. Rep..

[30] Akin-Ojo O., Szalewicz K. (2005). Potential energy surface and second virial coefficient of methane-water from ab initio calculations. J. Chem. Phys..

[31] Gu Y., Kar T., Scheiner S. (1999). Fundamental properties of the CH···O interaction: Is it a true hydrogen bond?. J. Am. Chem. Soc..

[32] Zrilić S.S., Živković J.M., Ninković D.B., Zarić S.D. (2025). Strong CH…O interactions in the second coordination sphere of 1,10-phenanthroline complexes with water. Int. J. Mol. Sci..

[33] Ninković D.B., Živković J.M., Zarić S.D. (2025). CH/O hydrogen bonds in the second coordination sphere of bipyridine complexes with water: Stronger than classical water-water hydrogen bonds. Inorg. Chem. Commun..

[34] Tarakeshwar P., Choi H.S., Kim K.S. (2001). Olefinic vs aromatic π−h interaction: A theoretical investigation of the nature of interaction of first-row hydrides with ethene and benzene. J. Am. Chem. Soc..

[35] Tsuzuki S., Uchimaru T. (2006). Magnitude and physical origin of intermolecular interactions of aromatic molecules: Recent progress of computational studies. Curr. Org. Chem..

[36] Tarakeshwar P., Choi H.S., Lee S.J., Lee J.Y., Kim K.S., Ha T.-K., Jang J.H., Lee J.G., Lee H. (1999). A theoretical investigation of the nature of the π-H interaction in ethene-H_2_O, benzene-H_2_O, and benzene-(H_2_O)_2_. J. Chem. Phys..

[37] Malenov D.P., Živković J.M., Zarić S.D. (2025). Hydrogen bonds in the second coordination sphere of metal complexes in the gas phase—Playing by the rules?. Dalton Trans..

[38] Vojislavljević D.Z., Janjić G.V., Ninković D.B., Kapor A., Zarić S.D. (2013). The influence of water molecule coordination onto the water-aromatic interaction. Strong interactions of water coordinating to a metal ion. CrystEngComm.

[39] Blagojević Filipović J.P., Vojislavljević-Vasilev D.Z., Zarić S.D. (2024). Crystallographic and quantum chemical study of NH/π interactions of metal ammine complexes with aromatic rings in the second coordination sphere. Cryst. Growth Des..

[40] Milovanović M.R., Stanković I.M., Živković J.M., Ninković D.B., Hall M.B., Zarić S.D. (2022). Water: New aspect of hydrogen bonding in the solid state. IUCrJ.

[41] Milovanović M.R., Živković J.M., Ninković D.B., Zarić S.D. (2023). Potential energy surfaces of antiparallel water-water interactions. J. Mol. Liq..

[42] Milovanović M.R., Zarić S.D. (2024). New aspects of alcohol–alcohol and alcohol–water interactions: Crystallographic and quantum chemical studies of antiparallel O–H/O–H interactions. J. Phys. Chem. Lett..

[43] Janjić G.V., Veljković D.Ž., Zarić S.D. (2011). Water/aromatic parallel alignment interactions. significant interactions at large horizontal displacements. Cryst. Growth Des..

[44] Vojislavljević-Vasilev D.Z., Janjić G.V., Medaković V.B., Blagojević J.P., Zarić S.D. (2014). Parallel water/aromatic interactions of non-coordinated and coordinated water. ChemPhysChem.

[45] Jeziorski B., Moszynski R., Szalewicz K. (1994). Perturbation theory approach to intermolecular potential energy surfaces of van der Waals complexes. Chem. Rev..

[46] Huang Y., Li K., Jiang X., Su Y., Cao X., Zhao J. (2018). Phase Diagram of Methane Hydrates and Discovery of MH-VI Hydrate. J. Phys. Chem..

[47] Blanco M.A., Martín Pendás A., Francisco E. (2005). Interacting Quantum Atoms: A Correlated Energy Decomposition Scheme Based on the Quantum Theory of Atoms in Molecules. J. Chem. Theory Comput..

[48] Guevara-Vela J.M., Francisco E., Rocha-Rinza T., Pendás Á.M. (2020). Interacting Quantum Atoms—A Review. Molecules.

[49] Maxwell P., Martín Pendás Á., Popelier P.L.A. (2016). Extension of the interacting quantum atoms (IQA) approach to B3LYP level density functional theory (DFT). Phys. Chem. Chem. Phys..

[50] Hohenstein E.G., Sherrill C.D. (2010). Density fitting of intramonomer correlation effects in symmetry-adapted perturbation theory. J. Chem. Phys..

[51] Szalewicz K., Jeziorski B. (2022). Physical mechanisms of intermolecular interactions from symmetry-adapted perturbation theory. J. Mol. Model.

[52] Rappoport D., Furche F. (2010). Property-optimized Gaussian basis sets for molecular response calculations. J. Chem. Phys..

[53] Parrish R.M., Burns L.A., Smith D.G.A., Simmonett A.C., DePrince A.E., Hohenstein E.G., Bozkaya U., Sokolov A.Y., Di Remigio R., Richard R.M. (2017). Psi4 1.1: An open-source electronic structure program emphasizing automation, advanced libraries, and interoperability. J. Chem. Theory Comput..

[54] Møller C., Plesset M.S. (1934). Note on an approximation treatment for many–electron systems. Phys. Rev..

[55] Kendall R.A., Dunning T.H., Harrison R.J. (1992). Electron affinities of the first–row atoms revisited. Systematic basis sets and wave functions. J. Chem. Phys..

[56] Becke A.D. (1993). Density-functional thermochemistry. III. The role of exact exchange. J. Chem. Phys..

[57] Lee C., Yang W., Parr R.G. (1988). Development of the Colle-Salvetti correlation-energy formula into a functional of the electron density. Phys. Rev. B..

[58] Grimme S., Ehrlich S., Goerigk L. (2011). Effect of the damping function in dispersion corrected density functional theory. J. Comput. Chem..

[59] Weigend F., Ahlrichs R. (2005). Balanced basis sets of split valence, triple zeta valence and quadruple zeta valence quality for H to Rn: Design and assessment of accuracy. Phys. Chem. Chem. Phys..

