# Supplementary material for: Influence of Small Changes in Intermolecular Distances on Results of Interaction Energy Decomposition for Stacking Interactions, Hydrogen Bonds, and Antiparallel Interactions
Source: Int J Mol Sci. 2026 Oct 23;27(21):9436. doi: 10.3390/ijms27219436 (PMC13762498; doi:10.3390/ijms27219436)
Supplement: Supplementary file 1 [file ijms-27-09436-s001.zip › ijms-4526663-Supplementary.pdf]

Supplementary information

The interaction energy decomposition can differ significantly for small changes in the optimal intermolecular distances: the case of stacking, hydrogen bonds, and dipole/dipole interactions

Jelena P. Blagojević Filipović [1], Snežana D. Zarić [2]
[1] Innovative Center of the Faculty of Chemistry, Studentski trg 12-16, 11000 Belgrade, Serbia
[2] University of Belgrade-Faculty of Chemistry, Studentski trg 12-16, 11000 Belgrade, Serbia
Corresponding author and email: Snežana D. Zarić, szaric@chem.bg.ac.rs

SI-I: Numerical tables of the SAPT energy components displayed in Figures 1-8

R	ELST.	EXCH-REP.	IND.	DISP.	NET DISP.	E int
Benzene/benzene, r=0.0 Å						
3.7	-0.62	5.93	-0.31	-6.64	-0.70	-1.64
3.8	-0.20	4.43	-0.26	-5.76	-1.33	-1.80
3.9	0.10	3.30	-0.22	-5.01	-1.71	-1.83
4.0	0.30	2.45	-0.19	-4.36	-1.91	-1.80
4.1	0.44	1.82	-0.16	-3.81	-1.99	-1.71
Benzene/benzene, r=1.5 Å						
3.3	-3.41	11.79	-1.16	-9.63	2.15	-2.42
3.4	-2.32	8.85	-0.91	-8.33	0.52	-2.71
3.5	-1.51	6.63	-0.71	-7.22	-0.59	-2.81
3.6	-0.92	4.96	-0.56	-6.27	-1.31	-2.79
3.7	-0.49	3.70	-0.45	-5.45	-1.75	-2.69
Benzene/benzene, r=5.0 Å						
2.4	-1.94	4.54	-0.48	-3.94	0.60	-1.81
2.5	-1.62	3.60	-0.38	-3.52	0.08	-1.92
2.6	-1.36	2.85	-0.31	-3.14	-0.30	-1.96
2.7	-1.14	2.25	-0.25	-2.81	-0.56	-1.95
2.8	-0.96	1.78	-0.20	-2.52	-0.74	-1.91
Benzene/pyridine, r=0.0 Å						
3.6	-1.48	7.05	-0.36	-7.28	-0.23	-2.07
3.7	-0.92	5.24	-0.30	-6.30	-1.05	-2.27
3.8	-0.52	3.89	-0.25	-5.46	-1.57	-2.33
3.9	-0.23	2.89	-0.21	-4.74	-1.86	-2.30
4.0	-0.03	2.14	-0.18	-4.13	-1.99	-2.20
Benzene/pyridine, r=1.5 Å						
3.3	-3.50	10.54	-1.05	-9.16	1.38	-3.16
3.4	-2.48	7.87	-0.82	-7.91	-0.04	-3.34
3.5	-1.73	5.87	-0.64	-6.84	-0.97	-3.35
3.6	-1.18	4.37	-0.50	-5.94	-1.57	-3.25
3.7	-0.78	3.25	-0.40	-5.16	-1.91	-3.09
Benzene/pyridine, r=5.0 Å						
2.4	-1.81	4.08	-0.44	-3.70	0.37	-1.87
2.5	-1.53	3.22	-0.35	-3.30	-0.08	-1.97
2.6	-1.30	2.55	-0.28	-2.95	-0.41	-1.99
2.7	-1.12	2.01	-0.23	-2.64	-0.63	-1.98
2.8	-0.96	1.58	-0.19	-2.37	-0.78	-1.93

R	ELST.	EXCH-REP.	IND.	DISP.	NET DISP.	E int
Pyridine/pyridine, r=0.0 Å						
3.5	-2.63	8.28	-0.46	-7.99	0.29	-2.79
3.6	-1.91	6.13	-0.37	-6.89	-0.75	-3.04
3.7	-1.39	4.53	-0.30	-5.95	-1.42	-3.11
3.8	-1.01	3.35	-0.25	-5.15	-1.81	-3.07
3.9	-0.74	2.47	-0.21	-4.47	-2.01	-2.95
Pyridine/pyridine, r=1.5 Å						
3.2	-5.00	12.43	-1.23	-10.06	2.37	-3.86
3.3	-3.75	9.24	-0.95	-8.66	0.59	-4.11
3.4	-2.82	6.86	-0.73	-7.47	-0.61	-4.16
3.5	-2.13	5.09	-0.57	-6.46	-1.37	-4.08
3.6	-1.62	3.76	-0.45	-5.60	-1.83	-3.90
Pyridine/pyridine, r=5.0 Å						
2.4	-1.77	3.59	-0.40	-3.46	0.13	-2.03
2.5	-1.53	2.83	-0.32	-3.09	-0.26	-2.11
2.6	-1.34	2.23	-0.26	-2.76	-0.53	-2.12
2.7	-1.18	1.76	-0.21	-2.47	-0.71	-2.10
2.8	-1.05	1.38	-0.17	-2.21	-0.83	-2.05
Benzene/cyclohexane, r=0.0 Å						
4.0	-3.28	9.08	-1.09	-7.66	1.42	-2.95
4.1	-2.47	6.74	-0.83	-6.59	0.15	-3.15
4.2	-1.87	4.99	-0.63	-5.68	-0.69	-3.19
4.3	-1.42	3.69	-0.48	-4.91	-1.22	-3.12
4.4	-1.08	2.72	-0.37	-4.25	-1.53	-2.98
Benzene/cyclohexane, r=1.5 Å						
3.9	-2.81	8.03	-0.88	-7.13	0.90	-2.78
4.0	-2.12	6.02	-0.68	-6.16	-0.15	-2.94
4.1	-1.60	4.49	-0.52	-5.33	-0.84	-2.97
4.2	-1.22	3.35	-0.41	-4.63	-1.28	-2.90
4.3	-0.93	2.50	-0.32	-4.02	-1.53	-2.77
Benzene/cyclohexane, r=5.0 Å						
2.8	-0.88	3.20	-0.30	-3.37	-0.18	-1.36
2.9	-0.69	2.56	-0.25	-3.03	-0.47	-1.40
3.0	-0.53	2.04	-0.20	-2.72	-0.68	-1.41
3.1	-0.41	1.62	-0.16	-2.44	-0.82	-1.39
3.2	-0.32	1.29	-0.13	-2.19	-0.91	-1.35

**R**	**ELST.**	**EXCH-REP.**	**IND.**	**DISP.**	**NET DISP.**	**E int**
OH/O						
1.8	-9.36	11.93	-3.47	-3.08	8.85	-3.99
1.9	-7.68	8.32	-2.51	-2.46	5.86	-4.33
2.0	-6.39	5.79	-1.83	-1.97	3.82	-4.39
2.1	-5.38	4.02	-1.34	-1.59	2.44	-4.28
2.2	-4.58	2.79	-1.00	-1.28	1.51	-4.07
CH/O						
2.5	-1.06	1.86	-0.40	-0.98	0.88	-0.58
2.6	-0.82	1.31	-0.31	-0.81	0.51	-0.63
2.7	-0.64	0.93	-0.24	-0.67	0.26	-0.63
2.8	-0.51	0.65	-0.19	-0.56	0.09	-0.61
2.9	-0.42	0.46	-0.15	-0.47	-0.01	-0.57
OH/π						
2.2	-3.33	6.26	-1.48	-4.24	2.02	-2.79
2.3	-2.87	4.64	-1.19	-3.61	1.03	-3.03
2.4	-2.50	3.43	-0.97	-3.07	0.36	-3.11
2.5	-2.20	2.52	-0.78	-2.62	-0.10	-3.08
2.6	-1.96	1.85	-0.64	-2.24	-0.38	-2.98
Anti OH/OH						
1.15	-7.44	7.84	-1.53	-2.60	5.24	-3.73
1.25	-6.90	6.68	-1.31	-2.34	4.34	-3.88
1.35	-6.36	5.63	-1.12	-2.10	3.53	-3.95
1.45	-5.83	4.71	-0.95	-1.88	2.84	-3.94
1.55	-5.33	3.91	-0.80	-1.67	2.24	-3.89

SI-II:Cartesian coordinates of the systems used for the SAPT calculations

Potential energy curves (r and R are defined by Figs. 1–8)

Benzene/benzene

r=0 Å; R=3.7 Å

C	-1.179992	1.036647	0.000000
C	0.036324	0.356353	0.000000
C	0.055330	-1.037154	0.000000
C	-2.377302	0.323433	0.000000
C	-2.358296	-1.070074	0.000000
C	-1.141980	-1.750368	0.000000
H	-1.194737	2.117677	0.000000
H	0.965151	0.909637	0.000000
H	0.998901	-1.564900	0.000000
H	-3.320873	0.851179	0.000000
H	-3.287123	-1.623358	0.000000
H	-1.127235	-2.831398	0.000000
C	-1.179992	1.036647	3.700000
C	0.036480	0.356446	3.700000
C	0.055805	-1.037420	3.700000
C	-2.377460	0.323522	3.700000
C	-2.358763	-1.070353	3.700000
C	-1.141970	-1.751094	3.700000
H	-1.194735	2.117547	3.700000
H	0.965195	0.909663	3.699947
H	0.999263	-1.565102	3.699841
H	-3.320918	0.851204	3.699947
H	-3.287479	-1.623570	3.699841
H	-1.127227	-2.831993	3.699788

r=0 Å; R=3.8 Å

C	-1.179992	1.036647	0.000000
C	0.036324	0.356353	0.000000
C	0.055330	-1.037154	0.000000
C	-2.377302	0.323433	0.000000
C	-2.358296	-1.070074	0.000000
C	-1.141980	-1.750368	0.000000
H	-1.194737	2.117677	0.000000
H	0.965151	0.909637	0.000000
H	0.998901	-1.564900	0.000000
H	-3.320873	0.851179	0.000000
H	-3.287123	-1.623358	0.000000
H	-1.127235	-2.831398	0.000000
C	-1.179992	1.036647	3.800000
C	0.036480	0.356446	3.800000
C	0.055805	-1.037420	3.800000
C	-2.377460	0.323522	3.800000
C	-2.358763	-1.070353	3.800000
C	-1.141970	-1.751094	3.800000
H	-1.194735	2.117547	3.800000
H	0.965195	0.909663	3.799948
H	0.999263	-1.565102	3.799845
H	-3.320918	0.851204	3.799948
H	-3.287479	-1.623570	3.799845
H	-1.127227	-2.831993	3.799794

r=0 Å; *R*=3.9 Å

C	-1.179992	1.036647	0.000000
C	0.036324	0.356353	0.000000
C	0.055330	-1.037154	0.000000
C	-2.377302	0.323433	0.000000
C	-2.358296	-1.070074	0.000000
C	-1.141980	-1.750368	0.000000
H	-1.194737	2.117677	0.000000
H	0.965151	0.909637	0.000000
H	0.998901	-1.564900	0.000000
H	-3.320873	0.851179	0.000000
H	-3.287123	-1.623358	0.000000
H	-1.127235	-2.831398	0.000000
C	-1.179992	1.036647	3.900000
C	0.036480	0.356446	3.900000
C	0.055805	-1.037420	3.900000
C	-2.377460	0.323522	3.900000
C	-2.358763	-1.070353	3.900000
C	-1.141970	-1.751094	3.900000
H	-1.194735	2.117547	3.900000
H	0.965195	0.909663	3.899950
H	0.999263	-1.565102	3.899849
H	-3.320918	0.851204	3.899950
H	-3.287479	-1.623570	3.899849
H	-1.127227	-2.831993	3.899799

r=0 Å; *R*=4.0 Å

C	-1.179992	1.036647	0.000000
C	0.036324	0.356353	0.000000
C	0.055330	-1.037154	0.000000
C	-2.377302	0.323433	0.000000
C	-2.358296	-1.070074	0.000000
C	-1.141980	-1.750368	0.000000
H	-1.194737	2.117677	0.000000
H	0.965151	0.909637	0.000000
H	0.998901	-1.564900	0.000000
H	-3.320873	0.851179	0.000000
H	-3.287123	-1.623358	0.000000
H	-1.127235	-2.831398	0.000000
C	-1.179992	1.036647	4.000000
C	0.036480	0.356446	4.000000
C	0.055805	-1.037420	4.000000
C	-2.377460	0.323522	4.000000
C	-2.358763	-1.070353	4.000000
C	-1.141970	-1.751094	4.000000
H	-1.194735	2.117547	4.000000
H	0.965195	0.909663	3.999951
H	0.999263	-1.565102	3.999853
H	-3.320918	0.851204	3.999951
H	-3.287479	-1.623570	3.999853
H	-1.127227	-2.831993	3.999804

r=0 Å; *R*=4.1 Å

C	-1.179992	1.036647	0.000000
C	0.036324	0.356353	0.000000
C	0.055330	-1.037154	0.000000
C	-2.377302	0.323433	0.000000
C	-2.358296	-1.070074	0.000000
C	-1.141980	-1.750368	0.000000
H	-1.194737	2.117677	0.000000
H	0.965151	0.909637	0.000000
H	0.998901	-1.564900	0.000000
H	-3.320873	0.851179	0.000000
H	-3.287123	-1.623358	0.000000
H	-1.127235	-2.831398	0.000000
C	-1.179992	1.036647	4.100000
C	0.036480	0.356446	4.100000
C	0.055805	-1.037420	4.100000
C	-2.377460	0.323522	4.100000
C	-2.358763	-1.070353	4.100000
C	-1.141970	-1.751094	4.100000
H	-1.194735	2.117547	4.100000
H	0.965195	0.909663	4.099952
H	0.999263	-1.565102	4.099856
H	-3.320918	0.851204	4.099952
H	-3.287479	-1.623570	4.099856
H	-1.127227	-2.831993	4.099809

r=1.5 Å; *R*=3.3 Å

C	-1.179992	1.036647	0.000000
C	0.036324	0.356353	0.000000
C	0.055330	-1.037154	0.000000
C	-2.377302	0.323433	0.000000
C	-2.358296	-1.070074	0.000000
C	-1.141980	-1.750368	0.000000
H	-1.194737	2.117677	0.000000
H	0.965151	0.909637	0.000000
H	0.998901	-1.564900	0.000000
H	-3.320873	0.851179	0.000000
H	-3.287123	-1.623358	0.000000
H	-1.127235	-2.831398	0.000000
C	0.319868	1.057104	3.300000
C	1.536501	0.376633	3.300000
C	1.555512	-1.017237	3.300000
C	0.357891	-1.730637	3.300000
C	-0.877753	0.343705	3.300000
C	-0.858742	-1.050166	3.300000
H	0.305126	2.138003	3.300000
H	2.465216	0.929850	3.300000
H	2.498970	-1.544920	3.300000
H	0.372633	-2.811536	3.300000
H	-1.821211	0.871387	3.300000
H	-1.787457	-1.603383	3.300000

r=1.5 Å; *R*=3.4 Å

C	-1.179992	1.036647	0.000000
C	0.036324	0.356353	0.000000
C	0.055330	-1.037154	0.000000
C	-2.377302	0.323433	0.000000
C	-2.358296	-1.070074	0.000000
C	-1.141980	-1.750368	0.000000
H	-1.194737	2.117677	0.000000
H	0.965151	0.909637	0.000000
H	0.998901	-1.564900	0.000000
H	-3.320873	0.851179	0.000000
H	-3.287123	-1.623358	0.000000
H	-1.127235	-2.831398	0.000000
C	0.319868	1.057104	3.400000
C	1.536501	0.376633	3.400000
C	1.555512	-1.017237	3.400000
C	0.357891	-1.730637	3.400000
C	-0.877753	0.343705	3.400000
C	-0.858742	-1.050166	3.400000
H	0.305126	2.138003	3.400000
H	2.465216	0.929850	3.400000
H	2.498970	-1.544920	3.400000
H	0.372633	-2.811536	3.400000
H	-1.821211	0.871387	3.400000
H	-1.787457	-1.603383	3.400000

r=1.5 Å; *R*=3.5 Å

C	-1.179992	1.036647	0.000000
C	0.036324	0.356353	0.000000
C	0.055330	-1.037154	0.000000
C	-2.377302	0.323433	0.000000
C	-2.358296	-1.070074	0.000000
C	-1.141980	-1.750368	0.000000
H	-1.194737	2.117677	0.000000
H	0.965151	0.909637	0.000000
H	0.998901	-1.564900	0.000000
H	-3.320873	0.851179	0.000000
H	-3.287123	-1.623358	0.000000
H	-1.127235	-2.831398	0.000000
C	0.319868	1.057104	3.500000
C	1.536501	0.376633	3.500000
C	1.555512	-1.017237	3.500000
C	0.357891	-1.730637	3.500000
C	-0.877753	0.343705	3.500000
C	-0.858742	-1.050166	3.500000
H	0.305126	2.138003	3.500000
H	2.465216	0.929850	3.500000
H	2.498970	-1.544920	3.500000
H	0.372633	-2.811536	3.500000
H	-1.821211	0.871387	3.500000
H	-1.787457	-1.603383	3.500000

r=1.5 Å; *R*=3.6 Å

C	-1.179992	1.036647	0.000000
C	0.036324	0.356353	0.000000
C	0.055330	-1.037154	0.000000
C	-2.377302	0.323433	0.000000
C	-2.358296	-1.070074	0.000000
C	-1.141980	-1.750368	0.000000
H	-1.194737	2.117677	0.000000
H	0.965151	0.909637	0.000000
H	0.998901	-1.564900	0.000000
H	-3.320873	0.851179	0.000000
H	-3.287123	-1.623358	0.000000
H	-1.127235	-2.831398	0.000000
C	0.319868	1.057104	3.600000
C	1.536501	0.376633	3.600000
C	1.555512	-1.017237	3.600000
C	0.357891	-1.730637	3.600000
C	-0.877753	0.343705	3.600000
C	-0.858742	-1.050166	3.600000
H	0.305126	2.138003	3.600000
H	2.465216	0.929850	3.600000
H	2.498970	-1.544920	3.600000
H	0.372633	-2.811536	3.600000
H	-1.821211	0.871387	3.600000
H	-1.787457	-1.603383	3.600000

r=1.5 Å; *R*=3.7 Å

C	-1.179992	1.036647	0.000000
C	0.036324	0.356353	0.000000
C	0.055330	-1.037154	0.000000
C	-2.377302	0.323433	0.000000
C	-2.358296	-1.070074	0.000000
C	-1.141980	-1.750368	0.000000
H	-1.194737	2.117677	0.000000
H	0.965151	0.909637	0.000000
H	0.998901	-1.564900	0.000000
H	-3.320873	0.851179	0.000000
H	-3.287123	-1.623358	0.000000
H	-1.127235	-2.831398	0.000000
C	0.319868	1.057104	3.700000
C	1.536501	0.376633	3.700000
C	1.555512	-1.017237	3.700000
C	0.357891	-1.730637	3.700000
C	-0.877753	0.343705	3.700000
C	-0.858742	-1.050166	3.700000
H	0.305126	2.138003	3.700000
H	2.465216	0.929850	3.700000
H	2.498970	-1.544920	3.700000
H	0.372633	-2.811536	3.700000
H	-1.821211	0.871387	3.700000
H	-1.787457	-1.603383	3.700000

r=5.0 Å; *R*=2.4 Å

C	-1.179992	1.036647	0.000000
C	0.036324	0.356353	0.000000
C	0.055330	-1.037154	0.000000
C	-2.377302	0.323433	0.000000
C	-2.358296	-1.070074	0.000000
C	-1.141980	-1.750368	0.000000
H	-1.194737	2.117677	0.000000
H	0.965151	0.909637	0.000000
H	0.998901	-1.564900	0.000000
H	-3.320873	0.851179	0.000000
H	-3.287123	-1.623358	0.000000
H	-1.127235	-2.831398	0.000000
C	3.819543	1.104836	-2.400000
C	5.036175	0.424365	-2.400000
C	5.055187	-0.969505	-2.400000
C	3.857565	-1.682904	-2.400000
C	2.621921	0.391437	-2.400000
C	2.640932	-1.002433	-2.400000
H	3.804800	2.185736	-2.400000
H	5.964891	0.977582	-2.400000
H	5.998644	-1.497187	-2.400000
H	3.872308	-2.763804	-2.400000
H	1.678464	0.919119	-2.400000
H	1.712217	-1.555650	-2.400000

r=5.0 Å; *R*=2.5 Å

C	-1.179992	1.036647	0.000000
C	0.036324	0.356353	0.000000
C	0.055330	-1.037154	0.000000
C	-2.377302	0.323433	0.000000
C	-2.358296	-1.070074	0.000000
C	-1.141980	-1.750368	0.000000
H	-1.194737	2.117677	0.000000
H	0.965151	0.909637	0.000000
H	0.998901	-1.564900	0.000000
H	-3.320873	0.851179	0.000000
H	-3.287123	-1.623358	0.000000
H	-1.127235	-2.831398	0.000000
C	3.819543	1.104836	-2.500000
C	5.036175	0.424365	-2.500000
C	5.055187	-0.969505	-2.500000
C	3.857565	-1.682904	-2.500000
C	2.621921	0.391437	-2.500000
C	2.640932	-1.002433	-2.500000
H	3.804800	2.185736	-2.500000
H	5.964891	0.977582	-2.500000
H	5.998644	-1.497187	-2.500000
H	3.872308	-2.763804	-2.500000
H	1.678464	0.919119	-2.500000
H	1.712217	-1.555650	-2.500000

r=5.0 Å; *R*=2.6 Å

C	-1.179992	1.036647	0.000000
C	0.036324	0.356353	0.000000
C	0.055330	-1.037154	0.000000
C	-2.377302	0.323433	0.000000
C	-2.358296	-1.070074	0.000000
C	-1.141980	-1.750368	0.000000
H	-1.194737	2.117677	0.000000
H	0.965151	0.909637	0.000000
H	0.998901	-1.564900	0.000000
H	-3.320873	0.851179	0.000000
H	-3.287123	-1.623358	0.000000
H	-1.127235	-2.831398	0.000000
C	3.819543	1.104836	-2.600000
C	5.036175	0.424365	-2.600000
C	5.055187	-0.969505	-2.600000
C	3.857565	-1.682904	-2.600000
C	2.621921	0.391437	-2.600000
C	2.640932	-1.002433	-2.600000
H	3.804800	2.185736	-2.600000
H	5.964891	0.977582	-2.600000
H	5.998644	-1.497187	-2.600000
H	3.872308	-2.763804	-2.600000
H	1.678464	0.919119	-2.600000
H	1.712217	-1.555650	-2.600000

r=5.0 Å; *R*=2.7 Å

C	-1.179992	1.036647	0.000000
C	0.036324	0.356353	0.000000
C	0.055330	-1.037154	0.000000
C	-2.377302	0.323433	0.000000
C	-2.358296	-1.070074	0.000000
C	-1.141980	-1.750368	0.000000
H	-1.194737	2.117677	0.000000
H	0.965151	0.909637	0.000000
H	0.998901	-1.564900	0.000000
H	-3.320873	0.851179	0.000000
H	-3.287123	-1.623358	0.000000
H	-1.127235	-2.831398	0.000000
C	3.819543	1.104836	-2.700000
C	5.036175	0.424365	-2.700000
C	5.055187	-0.969505	-2.700000
C	3.857565	-1.682904	-2.700000
C	2.621921	0.391437	-2.700000
C	2.640932	-1.002433	-2.700000
H	3.804800	2.185736	-2.700000
H	5.964891	0.977582	-2.700000
H	5.998644	-1.497187	-2.700000
H	3.872308	-2.763804	-2.700000
H	1.678464	0.919119	-2.700000
H	1.712217	-1.555650	-2.700000

r=5.0 Å; *R*=2.8 Å

C	-1.179992	1.036647	0.000000
C	0.036324	0.356353	0.000000
C	0.055330	-1.037154	0.000000
C	-2.377302	0.323433	0.000000
C	-2.358296	-1.070074	0.000000
C	-1.141980	-1.750368	0.000000
H	-1.194737	2.117677	0.000000
H	0.965151	0.909637	0.000000
H	0.998901	-1.564900	0.000000
H	-3.320873	0.851179	0.000000
H	-3.287123	-1.623358	0.000000
H	-1.127235	-2.831398	0.000000
C	3.819543	1.104836	-2.800000
C	5.036175	0.424365	-2.800000
C	5.055187	-0.969505	-2.800000
C	3.857565	-1.682904	-2.800000
C	2.621921	0.391437	-2.800000
C	2.640932	-1.002433	-2.800000
H	3.804800	2.185736	-2.800000
H	5.964891	0.977582	-2.800000
H	5.998644	-1.497187	-2.800000
H	3.872308	-2.763804	-2.800000
H	1.678464	0.919119	-2.800000
H	1.712217	-1.555650	-2.800000

Benzene/pyridine

r=0 Å; *R*=3.6 Å

C	-1.164655	1.030805	0.000000
C	0.059386	0.370091	0.000000
C	0.057496	-1.020885	0.000000
C	-2.332864	0.273379	0.000000
N	-2.352403	-1.066564	0.000000
C	-1.164833	-1.687457	0.000000
H	-1.218462	2.110125	0.000000
H	0.988244	0.923393	0.000000
H	0.980963	-1.582166	0.000000
H	-3.300278	0.760343	0.000000
H	-1.197304	-2.770031	0.000000
C	-2.347248	-1.063493	3.600000
C	-2.382954	0.299771	3.600000
C	-1.164797	1.043891	3.600000
C	0.047994	0.363306	3.600000
C	0.068818	-1.027177	3.600000
C	-1.165474	-1.743938	3.600000
H	-3.268949	-1.628319	3.600000
H	-3.332225	0.816923	3.600000
H	-1.178005	2.124810	3.600000
H	0.975969	0.917764	3.600000
H	1.006347	-1.565323	3.600000
H	-1.164201	-2.824937	3.600000

r=0 Å; *R*=3.7 Å

C	-1.164655	1.030805	0.000000
C	0.059386	0.370091	0.000000
C	0.057496	-1.020885	0.000000
C	-2.332864	0.273379	0.000000
N	-2.352403	-1.066564	0.000000
C	-1.164833	-1.687457	0.000000
H	-1.218462	2.110125	0.000000
H	0.988244	0.923393	0.000000
H	0.980963	-1.582166	0.000000
H	-3.300278	0.760343	0.000000
H	-1.197304	-2.770031	0.000000
C	-2.347248	-1.063493	3.700000
C	-2.382954	0.299771	3.700000
C	-1.164797	1.043891	3.700000
C	0.047994	0.363306	3.700000
C	0.068818	-1.027177	3.700000
C	-1.165474	-1.743938	3.700000
H	-3.268949	-1.628319	3.700000
H	-3.332225	0.816923	3.700000
H	-1.178005	2.124810	3.700000
H	0.975969	0.917764	3.700000
H	1.006347	-1.565323	3.700000
H	-1.164201	-2.824937	3.700000

r=0 Å; *R*=3.8 Å

C	-1.164655	1.030805	0.000000
C	0.059386	0.370091	0.000000
C	0.057496	-1.020885	0.000000
C	-2.332864	0.273379	0.000000
N	-2.352403	-1.066564	0.000000
C	-1.164833	-1.687457	0.000000
H	-1.218462	2.110125	0.000000
H	0.988244	0.923393	0.000000
H	0.980963	-1.582166	0.000000
H	-3.300278	0.760343	0.000000
H	-1.197304	-2.770031	0.000000
C	-2.347248	-1.063493	3.800000
C	-2.382954	0.299771	3.800000
C	-1.164797	1.043891	3.800000
C	0.047994	0.363306	3.800000
C	0.068818	-1.027177	3.800000
C	-1.165474	-1.743938	3.800000
H	-3.268949	-1.628319	3.800000
H	-3.332225	0.816923	3.800000
H	-1.178005	2.124810	3.800000
H	0.975969	0.917764	3.800000
H	1.006347	-1.565323	3.800000
H	-1.164201	-2.824937	3.800000

r=0 Å; *R*=3.9 Å

C	-1.164655	1.030805	0.000000
C	0.059386	0.370091	0.000000
C	0.057496	-1.020885	0.000000
C	-2.332864	0.273379	0.000000
N	-2.352403	-1.066564	0.000000
C	-1.164833	-1.687457	0.000000
H	-1.218462	2.110125	0.000000
H	0.988244	0.923393	0.000000
H	0.980963	-1.582166	0.000000
H	-3.300278	0.760343	0.000000
H	-1.197304	-2.770031	0.000000
C	-2.347248	-1.063493	3.900000
C	-2.382954	0.299771	3.900000
C	-1.164797	1.043891	3.900000
C	0.047994	0.363306	3.900000
C	0.068818	-1.027177	3.900000
C	-1.165474	-1.743938	3.900000
H	-3.268949	-1.628319	3.900000
H	-3.332225	0.816923	3.900000
H	-1.178005	2.124810	3.900000
H	0.975969	0.917764	3.900000
H	1.006347	-1.565323	3.900000
H	-1.164201	-2.824937	3.900000

r=0 Å; *R*=4.0 Å

C	-1.164655	1.030805	0.000000
C	0.059386	0.370091	0.000000
C	0.057496	-1.020885	0.000000
C	-2.332864	0.273379	0.000000
N	-2.352403	-1.066564	0.000000
C	-1.164833	-1.687457	0.000000
H	-1.218462	2.110125	0.000000
H	0.988244	0.923393	0.000000
H	0.980963	-1.582166	0.000000
H	-3.300278	0.760343	0.000000
H	-1.197304	-2.770031	0.000000
C	-2.347248	-1.063493	4.000000
C	-2.382954	0.299771	4.000000
C	-1.164797	1.043891	4.000000
C	0.047994	0.363306	4.000000
C	0.068818	-1.027177	4.000000
C	-1.165474	-1.743938	4.000000
H	-3.268949	-1.628319	4.000000
H	-3.332225	0.816923	4.000000
H	-1.178005	2.124810	4.000000
H	0.975969	0.917764	4.000000
H	1.006347	-1.565323	4.000000
H	-1.164201	-2.824937	4.000000

r=1.5 Å; *R*=3.3 Å

C	-1.164655	1.030805	0.000000
C	0.059386	0.370091	0.000000
C	0.057496	-1.020885	0.000000
C	-2.332864	0.273379	0.000000
N	-2.352403	-1.066564	0.000000
C	-1.164833	-1.687457	0.000000
H	-1.218462	2.110125	0.000000
H	0.988244	0.923393	0.000000
H	0.980963	-1.582166	0.000000
H	-3.300278	0.760343	0.000000
H	-1.197304	-2.770031	0.000000
C	-3.114966	0.225153	3.300000
C	-3.134055	1.619023	3.300000
C	-1.936473	2.332488	3.300000
C	-0.719802	1.652085	3.300000
C	-0.700714	0.258215	3.300000
C	-1.898296	-0.455250	3.300000
H	-4.043651	-0.328115	3.300000
H	-4.077542	2.146653	3.300000
H	-1.951275	3.413387	3.300000
H	0.208882	2.205353	3.300000
H	0.242773	-0.269415	3.300000
H	-1.883493	-1.536149	3.300000

r=1.5 Å; *R*=3.4 Å

C	-1.164655	1.030805	0.000000
C	0.059386	0.370091	0.000000
C	0.057496	-1.020885	0.000000
C	-2.332864	0.273379	0.000000
N	-2.352403	-1.066564	0.000000
C	-1.164833	-1.687457	0.000000
H	-1.218462	2.110125	0.000000
H	0.988244	0.923393	0.000000
H	0.980963	-1.582166	0.000000
H	-3.300278	0.760343	0.000000
H	-1.197304	-2.770031	0.000000
C	-3.114966	0.225153	3.400000
C	-3.134055	1.619023	3.400000
C	-1.936473	2.332488	3.400000
C	-0.719802	1.652085	3.400000
C	-0.700714	0.258215	3.400000
C	-1.898296	-0.455250	3.400000
H	-4.043651	-0.328115	3.400000
H	-4.077542	2.146653	3.400000
H	-1.951275	3.413387	3.400000
H	0.208882	2.205353	3.400000
H	0.242773	-0.269415	3.400000
H	-1.883493	-1.536149	3.400000

r=1.5 Å; *R*=3.5 Å

C	-1.164655	1.030805	0.000000
C	0.059386	0.370091	0.000000
C	0.057496	-1.020885	0.000000
C	-2.332864	0.273379	0.000000
N	-2.352403	-1.066564	0.000000
C	-1.164833	-1.687457	0.000000
H	-1.218462	2.110125	0.000000
H	0.988244	0.923393	0.000000
H	0.980963	-1.582166	0.000000
H	-3.300278	0.760343	0.000000
H	-1.197304	-2.770031	0.000000
C	-3.114966	0.225153	3.500000
C	-3.134055	1.619023	3.500000
C	-1.936473	2.332488	3.500000
C	-0.719802	1.652085	3.500000
C	-0.700714	0.258215	3.500000
C	-1.898296	-0.455250	3.500000
H	-4.043651	-0.328115	3.500000
H	-4.077542	2.146653	3.500000
H	-1.951275	3.413387	3.500000
H	0.208882	2.205353	3.500000
H	0.242773	-0.269415	3.500000
H	-1.883493	-1.536149	3.500000

r=1.5 Å; *R*=3.6 Å

C	-1.164655	1.030805	0.000000
C	0.059386	0.370091	0.000000
C	0.057496	-1.020885	0.000000
C	-2.332864	0.273379	0.000000
N	-2.352403	-1.066564	0.000000
C	-1.164833	-1.687457	0.000000
H	-1.218462	2.110125	0.000000
H	0.988244	0.923393	0.000000
H	0.980963	-1.582166	0.000000
H	-3.300278	0.760343	0.000000
H	-1.197304	-2.770031	0.000000
C	-3.114966	0.225153	3.600000
C	-3.134055	1.619023	3.600000
C	-1.936473	2.332488	3.600000
C	-0.719802	1.652085	3.600000
C	-0.700714	0.258215	3.600000
C	-1.898296	-0.455250	3.600000
H	-4.043651	-0.328115	3.600000
H	-4.077542	2.146653	3.600000
H	-1.951275	3.413387	3.600000
H	0.208882	2.205353	3.600000
H	0.242773	-0.269415	3.600000
H	-1.883493	-1.536149	3.600000

r=1.5 Å; *R*=3.7 Å

C	-1.164655	1.030805	0.000000
C	0.059386	0.370091	0.000000
C	0.057496	-1.020885	0.000000
C	-2.332864	0.273379	0.000000
N	-2.352403	-1.066564	0.000000
C	-1.164833	-1.687457	0.000000
H	-1.218462	2.110125	0.000000
H	0.988244	0.923393	0.000000
H	0.980963	-1.582166	0.000000
H	-3.300278	0.760343	0.000000
H	-1.197304	-2.770031	0.000000
C	-3.114966	0.225153	3.700000
C	-3.134055	1.619023	3.700000
C	-1.936473	2.332488	3.700000
C	-0.719802	1.652085	3.700000
C	-0.700714	0.258215	3.700000
C	-1.898296	-0.455250	3.700000
H	-4.043651	-0.328115	3.700000
H	-4.077542	2.146653	3.700000
H	-1.951275	3.413387	3.700000
H	0.208882	2.205353	3.700000
H	0.242773	-0.269415	3.700000
H	-1.883493	-1.536149	3.700000

r=5.0 Å; *R*=2.4 Å

C	-1.164655	1.030805	0.000000
C	0.059386	0.370091	0.000000
C	0.057496	-1.020885	0.000000
C	-2.332864	0.273379	0.000000
N	-2.352403	-1.066564	0.000000
C	-1.164833	-1.687457	0.000000
H	-1.218462	2.110125	0.000000
H	0.988244	0.923393	0.000000
H	0.980963	-1.582166	0.000000
H	-3.300278	0.760343	0.000000
H	-1.197304	-2.770031	0.000000
C	-4.906308	3.231995	-2.400000
C	-4.925396	4.625864	-2.400000
C	-3.727814	5.339330	-2.400000
C	-2.511144	4.658926	-2.400000
C	-2.492055	3.265057	-2.400000
C	-3.689637	2.551591	-2.400000
H	-5.834992	2.678726	-2.400000
H	-5.868883	5.153494	-2.400000
H	-3.742617	6.420228	-2.400000
H	-1.582459	5.212195	-2.400000
H	-1.548568	2.737427	-2.400000
H	-3.674835	1.470692	-2.400000

r=5.0 Å; *R*=2.5 Å

C	-1.164655	1.030805	0.000000
C	0.059386	0.370091	0.000000
C	0.057496	-1.020885	0.000000
C	-2.332864	0.273379	0.000000
N	-2.352403	-1.066564	0.000000
C	-1.164833	-1.687457	0.000000
H	-1.218462	2.110125	0.000000
H	0.988244	0.923393	0.000000
H	0.980963	-1.582166	0.000000
H	-3.300278	0.760343	0.000000
H	-1.197304	-2.770031	0.000000
C	-4.906308	3.231995	-2.500000
C	-4.925396	4.625864	-2.500000
C	-3.727814	5.339330	-2.500000
C	-2.511144	4.658926	-2.500000
C	-2.492055	3.265057	-2.500000
C	-3.689637	2.551591	-2.500000
H	-5.834992	2.678726	-2.500000
H	-5.868883	5.153494	-2.500000
H	-3.742617	6.420228	-2.500000
H	-1.582459	5.212195	-2.500000
H	-1.548568	2.737427	-2.500000
H	-3.674835	1.470692	-2.500000

r=5.0 Å; *R*=2.6 Å

C	-1.164655	1.030805	0.000000
C	0.059386	0.370091	0.000000
C	0.057496	-1.020885	0.000000
C	-2.332864	0.273379	0.000000
N	-2.352403	-1.066564	0.000000
C	-1.164833	-1.687457	0.000000
H	-1.218462	2.110125	0.000000
H	0.988244	0.923393	0.000000
H	0.980963	-1.582166	0.000000
H	-3.300278	0.760343	0.000000
H	-1.197304	-2.770031	0.000000
C	-4.906308	3.231995	-2.600000
C	-4.925396	4.625864	-2.600000
C	-3.727814	5.339330	-2.600000
C	-2.511144	4.658926	-2.600000
C	-2.492055	3.265057	-2.600000
C	-3.689637	2.551591	-2.600000
H	-5.834992	2.678726	-2.600000
H	-5.868883	5.153494	-2.600000
H	-3.742617	6.420228	-2.600000
H	-1.582459	5.212195	-2.600000
H	-1.548568	2.737427	-2.600000
H	-3.674835	1.470692	-2.600000

r=5.0 Å; *R*=2.7 Å

C	-1.164655	1.030805	0.000000
C	0.059386	0.370091	0.000000
C	0.057496	-1.020885	0.000000
C	-2.332864	0.273379	0.000000
N	-2.352403	-1.066564	0.000000
C	-1.164833	-1.687457	0.000000
H	-1.218462	2.110125	0.000000
H	0.988244	0.923393	0.000000
H	0.980963	-1.582166	0.000000
H	-3.300278	0.760343	0.000000
H	-1.197304	-2.770031	0.000000
C	-4.906308	3.231995	-2.700000
C	-4.925396	4.625864	-2.700000
C	-3.727814	5.339330	-2.700000
C	-2.511144	4.658926	-2.700000
C	-2.492055	3.265057	-2.700000
C	-3.689637	2.551591	-2.700000
H	-5.834992	2.678726	-2.700000
H	-5.868883	5.153494	-2.700000
H	-3.742617	6.420228	-2.700000
H	-1.582459	5.212195	-2.700000
H	-1.548568	2.737427	-2.700000
H	-3.674835	1.470692	-2.700000

r=5.0 Å; *R*=2.8 Å

C	-1.164655	1.030805	0.000000
C	0.059386	0.370091	0.000000
C	0.057496	-1.020885	0.000000
C	-2.332864	0.273379	0.000000
N	-2.352403	-1.066564	0.000000
C	-1.164833	-1.687457	0.000000
H	-1.218462	2.110125	0.000000
H	0.988244	0.923393	0.000000
H	0.980963	-1.582166	0.000000
H	-3.300278	0.760343	0.000000
H	-1.197304	-2.770031	0.000000
C	-4.906308	3.231995	-2.800000
C	-4.925396	4.625864	-2.800000
C	-3.727814	5.339330	-2.800000
C	-2.511144	4.658926	-2.800000
C	-2.492055	3.265057	-2.800000
C	-3.689637	2.551591	-2.800000
H	-5.834992	2.678726	-2.800000
H	-5.868883	5.153494	-2.800000
H	-3.742617	6.420228	-2.800000
H	-1.582459	5.212195	-2.800000
H	-1.548568	2.737427	-2.800000
H	-3.674835	1.470692	-2.800000

Pyridine/pyridine

r=0 Å; *R*=3.5 Å

C	-1.164655	1.030805	0.000000
C	0.059386	0.370091	0.000000
C	0.057496	-1.020885	0.000000
C	-2.332864	0.273379	0.000000
N	-2.352403	-1.066564	0.000000
C	-1.164833	-1.687457	0.000000
H	-1.218462	2.110125	0.000000
H	0.988244	0.923393	0.000000
H	0.980963	-1.582166	0.000000
H	-3.300278	0.760343	0.000000
H	-1.197304	-2.770031	0.000000
N	0.053070	0.366509	3.500000
C	-1.164241	0.986893	3.500000
C	-2.371437	0.293741	3.500000
C	-2.358417	-1.070147	3.500000
C	-1.165222	-1.730940	3.500000
C	0.019073	-0.999348	3.500000
H	-1.155617	2.069859	3.500000
H	-3.301293	0.845039	3.500000
H	-3.293056	-1.613295	3.500000
H	-1.134936	-2.811515	3.500000
H	0.981395	-1.496164	3.500000

r=0 Å; *R*=3.6 Å

C	-1.164655	1.030805	0.000000
C	0.059386	0.370091	0.000000
C	0.057496	-1.020885	0.000000
C	-2.332864	0.273379	0.000000
N	-2.352403	-1.066564	0.000000
C	-1.164833	-1.687457	0.000000
H	-1.218462	2.110125	0.000000
H	0.988244	0.923393	0.000000
H	0.980963	-1.582166	0.000000
H	-3.300278	0.760343	0.000000
H	-1.197304	-2.770031	0.000000
N	0.053070	0.366509	3.600000
C	-1.164241	0.986893	3.600000
C	-2.371437	0.293741	3.600000
C	-2.358417	-1.070147	3.600000
C	-1.165222	-1.730940	3.600000
C	0.019073	-0.999348	3.600000
H	-1.155617	2.069859	3.600000
H	-3.301293	0.845039	3.600000
H	-3.293056	-1.613295	3.600000
H	-1.134936	-2.811515	3.600000
H	0.981395	-1.496164	3.600000

r=0 Å; *R*=3.7 Å

C	-1.164655	1.030805	0.000000
C	0.059386	0.370091	0.000000
C	0.057496	-1.020885	0.000000
C	-2.332864	0.273379	0.000000
N	-2.352403	-1.066564	0.000000
C	-1.164833	-1.687457	0.000000
H	-1.218462	2.110125	0.000000
H	0.988244	0.923393	0.000000
H	0.980963	-1.582166	0.000000
H	-3.300278	0.760343	0.000000
H	-1.197304	-2.770031	0.000000
N	0.053070	0.366509	3.700000
C	-1.164241	0.986893	3.700000
C	-2.371437	0.293741	3.700000
C	-2.358417	-1.070147	3.700000
C	-1.165222	-1.730940	3.700000
C	0.019073	-0.999348	3.700000
H	-1.155617	2.069859	3.700000
H	-3.301293	0.845039	3.700000
H	-3.293056	-1.613295	3.700000
H	-1.134936	-2.811515	3.700000
H	0.981395	-1.496164	3.700000

r=0 Å; *R*=3.8 Å

C	-1.164655	1.030805	0.000000
C	0.059386	0.370091	0.000000
C	0.057496	-1.020885	0.000000
C	-2.332864	0.273379	0.000000
N	-2.352403	-1.066564	0.000000
C	-1.164833	-1.687457	0.000000
H	-1.218462	2.110125	0.000000
H	0.988244	0.923393	0.000000
H	0.980963	-1.582166	0.000000
H	-3.300278	0.760343	0.000000
H	-1.197304	-2.770031	0.000000
N	0.053070	0.366509	3.800000
C	-1.164241	0.986893	3.800000
C	-2.371437	0.293741	3.800000
C	-2.358417	-1.070147	3.800000
C	-1.165222	-1.730940	3.800000
C	0.019073	-0.999348	3.800000
H	-1.155617	2.069859	3.800000
H	-3.301293	0.845039	3.800000
H	-3.293056	-1.613295	3.800000
H	-1.134936	-2.811515	3.800000
H	0.981395	-1.496164	3.800000

r=0 Å; *R*=3.9 Å

C	-1.164655	1.030805	0.000000
C	0.059386	0.370091	0.000000
C	0.057496	-1.020885	0.000000
C	-2.332864	0.273379	0.000000
N	-2.352403	-1.066564	0.000000
C	-1.164833	-1.687457	0.000000
H	-1.218462	2.110125	0.000000
H	0.988244	0.923393	0.000000
H	0.980963	-1.582166	0.000000
H	-3.300278	0.760343	0.000000
H	-1.197304	-2.770031	0.000000
N	0.053070	0.366509	3.900000
C	-1.164241	0.986893	3.900000
C	-2.371437	0.293741	3.900000
C	-2.358417	-1.070147	3.900000
C	-1.165222	-1.730940	3.900000
C	0.019073	-0.999348	3.900000
H	-1.155617	2.069859	3.900000
H	-3.301293	0.845039	3.900000
H	-3.293056	-1.613295	3.900000
H	-1.134936	-2.811515	3.900000
H	0.981395	-1.496164	3.900000

r=1.5 Å; *R*=3.2 Å

C	-1.164655	1.030805	0.000000
C	0.059386	0.370091	0.000000
C	0.057496	-1.020885	0.000000
C	-2.332864	0.273379	0.000000
N	-2.352403	-1.066564	0.000000
C	-1.164833	-1.687457	0.000000
H	-1.218462	2.110125	0.000000
H	0.988244	0.923393	0.000000
H	0.980963	-1.582166	0.000000
H	-3.300278	0.760343	0.000000
H	-1.197304	-2.770031	0.000000
N	-0.714648	1.655156	3.200000
C	-1.931959	2.275540	3.200000
C	-3.139155	1.582388	3.200000
C	-3.126134	0.218500	3.200000
C	-1.932940	-0.442293	3.200000
C	-0.748645	0.289298	3.200000
H	-1.923335	3.358505	3.200000
H	-4.069011	2.133685	3.200000
H	-4.060774	-0.324649	3.200000
H	-1.902654	-1.522869	3.200000
H	0.213677	-0.207518	3.200000

r=1.5 Å; *R*=3.3 Å

C	-1.164655	1.030805	0.000000
C	0.059386	0.370091	0.000000
C	0.057496	-1.020885	0.000000
C	-2.332864	0.273379	0.000000
N	-2.352403	-1.066564	0.000000
C	-1.164833	-1.687457	0.000000
H	-1.218462	2.110125	0.000000
H	0.988244	0.923393	0.000000
H	0.980963	-1.582166	0.000000
H	-3.300278	0.760343	0.000000
H	-1.197304	-2.770031	0.000000
N	-0.714648	1.655156	3.300000
C	-1.931959	2.275540	3.300000
C	-3.139155	1.582388	3.300000
C	-3.126134	0.218500	3.300000
C	-1.932940	-0.442293	3.300000
C	-0.748645	0.289298	3.300000
H	-1.923335	3.358505	3.300000
H	-4.069011	2.133685	3.300000
H	-4.060774	-0.324649	3.300000
H	-1.902654	-1.522869	3.300000
H	0.213677	-0.207518	3.300000

r=1.5 Å; *R*=3.4 Å

C	-1.164655	1.030805	0.000000
C	0.059386	0.370091	0.000000
C	0.057496	-1.020885	0.000000
C	-2.332864	0.273379	0.000000
N	-2.352403	-1.066564	0.000000
C	-1.164833	-1.687457	0.000000
H	-1.218462	2.110125	0.000000
H	0.988244	0.923393	0.000000
H	0.980963	-1.582166	0.000000
H	-3.300278	0.760343	0.000000
H	-1.197304	-2.770031	0.000000
N	-0.714648	1.655156	3.400000
C	-1.931959	2.275540	3.400000
C	-3.139155	1.582388	3.400000
C	-3.126134	0.218500	3.400000
C	-1.932940	-0.442293	3.400000
C	-0.748645	0.289298	3.400000
H	-1.923335	3.358505	3.400000
H	-4.069011	2.133685	3.400000
H	-4.060774	-0.324649	3.400000
H	-1.902654	-1.522869	3.400000
H	0.213677	-0.207518	3.400000

r=1.5 Å; *R*=3.5 Å

C	-1.164655	1.030805	0.000000
C	0.059386	0.370091	0.000000
C	0.057496	-1.020885	0.000000
C	-2.332864	0.273379	0.000000
N	-2.352403	-1.066564	0.000000
C	-1.164833	-1.687457	0.000000
H	-1.218462	2.110125	0.000000
H	0.988244	0.923393	0.000000
H	0.980963	-1.582166	0.000000
H	-3.300278	0.760343	0.000000
H	-1.197304	-2.770031	0.000000
N	-0.714648	1.655156	3.500000
C	-1.931959	2.275540	3.500000
C	-3.139155	1.582388	3.500000
C	-3.126134	0.218500	3.500000
C	-1.932940	-0.442293	3.500000
C	-0.748645	0.289298	3.500000
H	-1.923335	3.358505	3.500000
H	-4.069011	2.133685	3.500000
H	-4.060774	-0.324649	3.500000
H	-1.902654	-1.522869	3.500000
H	0.213677	-0.207518	3.500000

r=1.5 Å; *R*=3.6 Å

C	-1.164655	1.030805	0.000000
C	0.059386	0.370091	0.000000
C	0.057496	-1.020885	0.000000
C	-2.332864	0.273379	0.000000
N	-2.352403	-1.066564	0.000000
C	-1.164833	-1.687457	0.000000
H	-1.218462	2.110125	0.000000
H	0.988244	0.923393	0.000000
H	0.980963	-1.582166	0.000000
H	-3.300278	0.760343	0.000000
H	-1.197304	-2.770031	0.000000
N	-0.714648	1.655156	3.600000
C	-1.931959	2.275540	3.600000
C	-3.139155	1.582388	3.600000
C	-3.126134	0.218500	3.600000
C	-1.932940	-0.442293	3.600000
C	-0.748645	0.289298	3.600000
H	-1.923335	3.358505	3.600000
H	-4.069011	2.133685	3.600000
H	-4.060774	-0.324649	3.600000
H	-1.902654	-1.522869	3.600000
H	0.213677	-0.207518	3.600000

r=5.0 Å; *R*=2.4 Å

C	-1.164655	1.030805	0.000000
C	0.059386	0.370091	0.000000
C	0.057496	-1.020885	0.000000
C	-2.332864	0.273379	0.000000
N	-2.352403	-1.066564	0.000000
C	-1.164833	-1.687457	0.000000
H	-1.218462	2.110125	0.000000
H	0.988244	0.923393	0.000000
H	0.980963	-1.582166	0.000000
H	-3.300278	0.760343	0.000000
H	-1.197304	-2.770031	0.000000
N	-2.505989	4.661997	2.400000
C	-3.723300	5.282381	2.400000
C	-4.930496	4.589229	2.400000
C	-4.917476	3.225341	2.400000
C	-3.724282	2.564548	2.400000
C	-2.539987	3.296139	2.400000
H	-3.714676	6.365347	2.400000
H	-5.860352	5.140527	2.400000
H	-5.852115	2.682192	2.400000
H	-3.693995	1.483972	2.400000
H	-1.577665	2.799324	2.400000

r=5.0 Å; *R*=2.5 Å

C	-1.164655	1.030805	0.000000
C	0.059386	0.370091	0.000000
C	0.057496	-1.020885	0.000000
C	-2.332864	0.273379	0.000000
N	-2.352403	-1.066564	0.000000
C	-1.164833	-1.687457	0.000000
H	-1.218462	2.110125	0.000000
H	0.988244	0.923393	0.000000
H	0.980963	-1.582166	0.000000
H	-3.300278	0.760343	0.000000
H	-1.197304	-2.770031	0.000000
N	-2.505989	4.661997	2.500000
C	-3.723300	5.282381	2.500000
C	-4.930496	4.589229	2.500000
C	-4.917476	3.225341	2.500000
C	-3.724282	2.564548	2.500000
C	-2.539987	3.296139	2.500000
H	-3.714676	6.365347	2.500000
H	-5.860352	5.140527	2.500000
H	-5.852115	2.682192	2.500000
H	-3.693995	1.483972	2.500000
H	-1.577665	2.799324	2.500000

r=5.0 Å; *R*=2.6 Å

C	-1.164655	1.030805	0.000000
C	0.059386	0.370091	0.000000
C	0.057496	-1.020885	0.000000
C	-2.332864	0.273379	0.000000
N	-2.352403	-1.066564	0.000000
C	-1.164833	-1.687457	0.000000
H	-1.218462	2.110125	0.000000
H	0.988244	0.923393	0.000000
H	0.980963	-1.582166	0.000000
H	-3.300278	0.760343	0.000000
H	-1.197304	-2.770031	0.000000
N	-2.505989	4.661997	2.600000
C	-3.723300	5.282381	2.600000
C	-4.930496	4.589229	2.600000
C	-4.917476	3.225341	2.600000
C	-3.724282	2.564548	2.600000
C	-2.539987	3.296139	2.600000
H	-3.714676	6.365347	2.600000
H	-5.860352	5.140527	2.600000
H	-5.852115	2.682192	2.600000
H	-3.693995	1.483972	2.600000
H	-1.577665	2.799324	2.600000

r=5.0 Å; *R*=2.7 Å

C	-1.164655	1.030805	0.000000
C	0.059386	0.370091	0.000000
C	0.057496	-1.020885	0.000000
C	-2.332864	0.273379	0.000000
N	-2.352403	-1.066564	0.000000
C	-1.164833	-1.687457	0.000000
H	-1.218462	2.110125	0.000000
H	0.988244	0.923393	0.000000
H	0.980963	-1.582166	0.000000
H	-3.300278	0.760343	0.000000
H	-1.197304	-2.770031	0.000000
N	-2.505989	4.661997	2.700000
C	-3.723300	5.282381	2.700000
C	-4.930496	4.589229	2.700000
C	-4.917476	3.225341	2.700000
C	-3.724282	2.564548	2.700000
C	-2.539987	3.296139	2.700000
H	-3.714676	6.365347	2.700000
H	-5.860352	5.140527	2.700000
H	-5.852115	2.682192	2.700000
H	-3.693995	1.483972	2.700000
H	-1.577665	2.799324	2.700000

r=5.0 Å; *R*=2.8 Å

C	-1.164655	1.030805	0.000000
C	0.059386	0.370091	0.000000
C	0.057496	-1.020885	0.000000
C	-2.332864	0.273379	0.000000
N	-2.352403	-1.066564	0.000000
C	-1.164833	-1.687457	0.000000
H	-1.218462	2.110125	0.000000
H	0.988244	0.923393	0.000000
H	0.980963	-1.582166	0.000000
H	-3.300278	0.760343	0.000000
H	-1.197304	-2.770031	0.000000
N	-2.505989	4.661997	2.800000
C	-3.723300	5.282381	2.800000
C	-4.930496	4.589229	2.800000
C	-4.917476	3.225341	2.800000
C	-3.724282	2.564548	2.800000
C	-2.539987	3.296139	2.800000
H	-3.714676	6.365347	2.800000
H	-5.860352	5.140527	2.800000
H	-5.852115	2.682192	2.800000
H	-3.693995	1.483972	2.800000
H	-1.577665	2.799324	2.800000

Benzene/cyclohexane

r=0 Å; *R*=4.0 Å

C	3.333373	-0.613814	1.997034
C	4.187244	-0.756089	0.739939
C	5.657221	-0.957340	1.098177
C	5.835063	-2.162068	2.018316
C	4.981189	-2.019793	3.275415
C	3.511216	-1.818547	2.917177
H	4.068573	0.118616	0.098775
H	6.026861	-0.061838	1.606598
H	6.255743	-1.077951	0.194017
H	6.885904	-2.285245	2.284477
H	5.534765	-3.067823	1.483079
H	5.330662	-1.155998	3.848803
H	5.099872	-2.894498	3.916578
H	3.141575	-2.714047	2.408752
H	2.912683	-1.697932	3.821329
H	3.837773	-1.619883	0.166548
H	3.633668	0.291937	2.532280
H	2.282529	-0.490643	1.730884
C	2.381263	-4.868642	1.019898
C	3.652168	-5.428346	1.141427
C	4.612117	-5.198280	0.157149
C	4.301163	-4.408510	-0.948657
C	3.030258	-3.848806	-1.070185
C	2.070308	-4.078872	-0.085908
H	1.636855	-5.047050	1.783173
H	3.893303	-6.040785	1.998942
H	5.597661	-5.632311	0.251390
H	5.045571	-4.230102	-1.711931
H	2.789123	-3.236367	-1.927701
H	1.084765	-3.644840	-0.180149

r=0 Å; *R*=4.1 Å

C	3.333373	-0.613814	1.997034
C	4.187244	-0.756089	0.739939
C	5.657221	-0.957340	1.098177
C	5.835063	-2.162068	2.018316
C	4.981189	-2.019793	3.275415
C	3.511216	-1.818547	2.917177
H	4.068573	0.118616	0.098775
H	6.026861	-0.061838	1.606598
H	6.255743	-1.077951	0.194017
H	6.885904	-2.285245	2.284477
H	5.534765	-3.067823	1.483079
H	5.330662	-1.155998	3.848803
H	5.099872	-2.894498	3.916578
H	3.141575	-2.714047	2.408752
H	2.912683	-1.697932	3.821329
H	3.837773	-1.619883	0.166548
H	3.633668	0.291937	2.532280
H	2.282529	-0.490643	1.730884
C	2.350189	-4.949906	0.970598
C	3.621094	-5.509609	1.092126
C	4.581043	-5.279544	0.107848
C	4.270088	-4.489774	-0.997958
C	2.999184	-3.930070	-1.119486
C	2.039234	-4.160136	-0.135208
H	1.605781	-5.128314	1.733872
H	3.862229	-6.122049	1.949641
H	5.566587	-5.713575	0.202090
H	5.014497	-4.311366	-1.761232
H	2.758049	-3.317631	-1.977001
H	1.053691	-3.726104	-0.229450

r=0 Å; *R*=4.2 Å

C	3.333373	-0.613814	1.997034
C	4.187244	-0.756089	0.739939
C	5.657221	-0.957340	1.098177
C	5.835063	-2.162068	2.018316
C	4.981189	-2.019793	3.275415
C	3.511216	-1.818547	2.917177
H	4.068573	0.118616	0.098775
H	6.026861	-0.061838	1.606598
H	6.255743	-1.077951	0.194017
H	6.885904	-2.285245	2.284477
H	5.534765	-3.067823	1.483079
H	5.330662	-1.155998	3.848803
H	5.099872	-2.894498	3.916578
H	3.141575	-2.714047	2.408752
H	2.912683	-1.697932	3.821329
H	3.837773	-1.619883	0.166548
H	3.633668	0.291937	2.532280
H	2.282529	-0.490643	1.730884
C	2.319115	-5.031169	0.921297
C	3.590020	-5.590873	1.042825
C	4.549969	-5.360808	0.058548
C	4.239014	-4.571038	-1.047258
C	2.968110	-4.011334	-1.168787
C	2.008160	-4.241400	-0.184509
H	1.574706	-5.209578	1.684571
H	3.831155	-6.203313	1.900341
H	5.535513	-5.794839	0.152789
H	4.983423	-4.392630	-1.810533
H	2.726975	-3.398895	-2.026302
H	1.022616	-3.807368	-0.278750

r=0 Å; *R*=4.3 Å

C	3.333373	-0.613814	1.997034
C	4.187244	-0.756089	0.739939
C	5.657221	-0.957340	1.098177
C	5.835063	-2.162068	2.018316
C	4.981189	-2.019793	3.275415
C	3.511216	-1.818547	2.917177
H	4.068573	0.118616	0.098775
H	6.026861	-0.061838	1.606598
H	6.255743	-1.077951	0.194017
H	6.885904	-2.285245	2.284477
H	5.534765	-3.067823	1.483079
H	5.330662	-1.155998	3.848803
H	5.099872	-2.894498	3.916578
H	3.141575	-2.714047	2.408752
H	2.912683	-1.697932	3.821329
H	3.837773	-1.619883	0.166548
H	3.633668	0.291937	2.532280
H	2.282529	-0.490643	1.730884
C	2.288041	-5.112433	0.871996
C	3.558945	-5.672137	0.993524
C	4.518895	-5.442072	0.009247
C	4.207940	-4.652302	-1.096559
C	2.937035	-4.092598	-1.218088
C	1.977086	-4.322664	-0.233810
H	1.543632	-5.290842	1.635270
H	3.800080	-6.284577	1.851040
H	5.504439	-5.876103	0.103488
H	4.952349	-4.473894	-1.859833
H	2.695900	-3.480159	-2.075603
H	0.991542	-3.888632	-0.328051

r=0 Å; *R*=4.4 Å

C	3.333373	-0.613814	1.997034
C	4.187244	-0.756089	0.739939
C	5.657221	-0.957340	1.098177
C	5.835063	-2.162068	2.018316
C	4.981189	-2.019793	3.275415
C	3.511216	-1.818547	2.917177
H	4.068573	0.118616	0.098775
H	6.026861	-0.061838	1.606598
H	6.255743	-1.077951	0.194017
H	6.885904	-2.285245	2.284477
H	5.534765	-3.067823	1.483079
H	5.330662	-1.155998	3.848803
H	5.099872	-2.894498	3.916578
H	3.141575	-2.714047	2.408752
H	2.912683	-1.697932	3.821329
H	3.837773	-1.619883	0.166548
H	3.633668	0.291937	2.532280
H	2.282529	-0.490643	1.730884
C	2.256967	-5.193697	0.822695
C	3.527871	-5.753401	0.944224
C	4.487821	-5.523336	-0.040054
C	4.176866	-4.733566	-1.145860
C	2.905961	-4.173862	-1.267388
C	1.946012	-4.403928	-0.283111
H	1.512558	-5.372105	1.585969
H	3.769006	-6.365841	1.801739
H	5.473364	-5.957367	0.054187
H	4.921274	-4.555158	-1.909134
H	2.664826	-3.561422	-2.124904
H	0.960468	-3.969896	-0.377352

r=1.5 Å; *R*=3.9 Å

C	3.333373	-0.613814	1.997034
C	4.187244	-0.756089	0.739939
C	5.657221	-0.957340	1.098177
C	5.835063	-2.162068	2.018316
C	4.981189	-2.019793	3.275415
C	3.511216	-1.818547	2.917177
H	4.068573	0.118616	0.098775
H	6.026861	-0.061838	1.606598
H	6.255743	-1.077951	0.194017
H	6.885904	-2.285245	2.284477
H	5.534765	-3.067823	1.483079
H	5.330662	-1.155998	3.848803
H	5.099872	-2.894498	3.916578
H	3.141575	-2.714047	2.408752
H	2.912683	-1.697932	3.821329
H	3.837773	-1.619883	0.166548
H	3.633668	0.291937	2.532280
H	2.282529	-0.490643	1.730884
C	3.349504	-3.488880	-1.661838
C	2.078600	-2.929176	-1.783366
C	1.118650	-3.159242	-0.799089
C	1.429605	-3.949010	0.306717
C	2.700509	-4.508714	0.428246
C	3.660459	-4.278650	-0.556032
H	4.093913	-3.310472	-2.425112
H	1.837464	-2.316736	-2.640882
H	0.133106	-2.725210	-0.893330
H	0.685196	-4.127418	1.069992
H	2.941644	-5.121154	1.285761
H	4.646003	-4.712680	-0.461791

r=1.5 Å; *R*=4.0 Å

C	3.333373	-0.613814	1.997034
C	4.187244	-0.756089	0.739939
C	5.657221	-0.957340	1.098177
C	5.835063	-2.162068	2.018316
C	4.981189	-2.019793	3.275415
C	3.511216	-1.818547	2.917177
H	4.068573	0.118616	0.098775
H	6.026861	-0.061838	1.606598
H	6.255743	-1.077951	0.194017
H	6.885904	-2.285245	2.284477
H	5.534765	-3.067823	1.483079
H	5.330662	-1.155998	3.848803
H	5.099872	-2.894498	3.916578
H	3.141575	-2.714047	2.408752
H	2.912683	-1.697932	3.821329
H	3.837773	-1.619883	0.166548
H	3.633668	0.291937	2.532280
H	2.282529	-0.490643	1.730884
C	3.318429	-3.570147	-1.711141
C	2.047525	-3.010443	-1.832669
C	1.087575	-3.240509	-0.848392
C	1.398530	-4.030278	0.257414
C	2.669434	-4.589982	0.378943
C	3.629384	-4.359917	-0.605335
H	4.062838	-3.391739	-2.474415
H	1.806389	-2.398003	-2.690185
H	0.102031	-2.806477	-0.942633
H	0.654121	-4.208686	1.020689
H	2.910569	-5.202422	1.236458
H	4.614928	-4.793948	-0.511094

r=1.5 Å; *R*=4.1 Å

C	3.333373	-0.613814	1.997034
C	4.187244	-0.756089	0.739939
C	5.657221	-0.957340	1.098177
C	5.835063	-2.162068	2.018316
C	4.981189	-2.019793	3.275415
C	3.511216	-1.818547	2.917177
H	4.068573	0.118616	0.098775
H	6.026861	-0.061838	1.606598
H	6.255743	-1.077951	0.194017
H	6.885904	-2.285245	2.284477
H	5.534765	-3.067823	1.483079
H	5.330662	-1.155998	3.848803
H	5.099872	-2.894498	3.916578
H	3.141575	-2.714047	2.408752
H	2.912683	-1.697932	3.821329
H	3.837773	-1.619883	0.166548
H	3.633668	0.291937	2.532280
H	2.282529	-0.490643	1.730884
C	3.287355	-3.651411	-1.760442
C	2.016450	-3.091707	-1.881970
C	1.056501	-3.321773	-0.897692
C	1.367456	-4.111542	0.208114
C	2.638360	-4.671246	0.329642
C	3.598310	-4.441181	-0.654636
H	4.031763	-3.473003	-2.523716
H	1.775315	-2.479267	-2.739485
H	0.070957	-2.887741	-0.991934
H	0.623047	-4.289950	0.971388
H	2.879495	-5.283686	1.187157
H	4.583853	-4.875212	-0.560395

r=1.5 Å; *R*=4.2 Å

C	3.333373	-0.613814	1.997034
C	4.187244	-0.756089	0.739939
C	5.657221	-0.957340	1.098177
C	5.835063	-2.162068	2.018316
C	4.981189	-2.019793	3.275415
C	3.511216	-1.818547	2.917177
H	4.068573	0.118616	0.098775
H	6.026861	-0.061838	1.606598
H	6.255743	-1.077951	0.194017
H	6.885904	-2.285245	2.284477
H	5.534765	-3.067823	1.483079
H	5.330662	-1.155998	3.848803
H	5.099872	-2.894498	3.916578
H	3.141575	-2.714047	2.408752
H	2.912683	-1.697932	3.821329
H	3.837773	-1.619883	0.166548
H	3.633668	0.291937	2.532280
H	2.282529	-0.490643	1.730884
C	3.256281	-3.732675	-1.809743
C	1.985376	-3.172971	-1.931271
C	1.025427	-3.403037	-0.946993
C	1.336382	-4.192806	0.158813
C	2.607286	-4.752510	0.280341
C	3.567236	-4.522445	-0.703937
H	4.000689	-3.554267	-2.573017
H	1.744241	-2.560531	-2.788786
H	0.039883	-2.969005	-1.041234
H	0.591973	-4.371214	0.922087
H	2.848421	-5.364950	1.137857
H	4.552779	-4.956476	-0.609695

r=1.5 Å; *R*=4.3 Å

C	3.333373	-0.613814	1.997034
C	4.187244	-0.756089	0.739939
C	5.657221	-0.957340	1.098177
C	5.835063	-2.162068	2.018316
C	4.981189	-2.019793	3.275415
C	3.511216	-1.818547	2.917177
H	4.068573	0.118616	0.098775
H	6.026861	-0.061838	1.606598
H	6.255743	-1.077951	0.194017
H	6.885904	-2.285245	2.284477
H	5.534765	-3.067823	1.483079
H	5.330662	-1.155998	3.848803
H	5.099872	-2.894498	3.916578
H	3.141575	-2.714047	2.408752
H	2.912683	-1.697932	3.821329
H	3.837773	-1.619883	0.166548
H	3.633668	0.291937	2.532280
H	2.282529	-0.490643	1.730884
C	3.225207	-3.813939	-1.859043
C	1.954302	-3.254235	-1.980572
C	0.994352	-3.484301	-0.996294
C	1.305307	-4.274070	0.109512
C	2.576212	-4.833774	0.231040
C	3.536161	-4.603708	-0.753237
H	3.969615	-3.635531	-2.622318
H	1.713167	-2.641795	-2.838087
H	0.008809	-3.050269	-1.090535
H	0.560899	-4.452478	0.872786
H	2.817347	-5.446214	1.088556
H	4.521705	-5.037740	-0.658996

r=5.0 Å; *R*=2.8 Å

C	3.333373	-0.613814	1.997034
C	4.187244	-0.756089	0.739939
C	5.657221	-0.957340	1.098177
C	5.835063	-2.162068	2.018316
C	4.981189	-2.019793	3.275415
C	3.511216	-1.818547	2.917177
H	4.068573	0.118616	0.098775
H	6.026861	-0.061838	1.606598
H	6.255743	-1.077951	0.194017
H	6.885904	-2.285245	2.284477
H	5.534765	-3.067823	1.483079
H	5.330662	-1.155998	3.848803
H	5.099872	-2.894498	3.916578
H	3.141575	-2.714047	2.408752
H	2.912683	-1.697932	3.821329
H	3.837773	-1.619883	0.166548
H	3.633668	0.291937	2.532280
H	2.282529	-0.490643	1.730884
C	1.398274	-0.638799	-2.898661
C	0.127370	-0.079095	-3.020189
C	-0.832580	-0.309161	-2.035912
C	-0.521625	-1.098930	-0.930106
C	0.749280	-1.658634	-0.808577
C	1.709229	-1.428569	-1.792855
H	2.142683	-0.460391	-3.661935
H	-0.113765	0.533345	-3.877705
H	-1.818123	0.124871	-2.130153
H	-1.266033	-1.277338	-0.166831
H	0.990415	-2.271074	0.048938
H	2.694773	-1.862600	-1.698614

r=5.0 Å; *R*=2.9 Å

C	3.333373	-0.613814	1.997034
C	4.187244	-0.756089	0.739939
C	5.657221	-0.957340	1.098177
C	5.835063	-2.162068	2.018316
C	4.981189	-2.019793	3.275415
C	3.511216	-1.818547	2.917177
H	4.068573	0.118616	0.098775
H	6.026861	-0.061838	1.606598
H	6.255743	-1.077951	0.194017
H	6.885904	-2.285245	2.284477
H	5.534765	-3.067823	1.483079
H	5.330662	-1.155998	3.848803
H	5.099872	-2.894498	3.916578
H	3.141575	-2.714047	2.408752
H	2.912683	-1.697932	3.821329
H	3.837773	-1.619883	0.166548
H	3.633668	0.291937	2.532280
H	2.282529	-0.490643	1.730884
C	1.367200	-0.720063	-2.947962
C	0.096296	-0.160359	-3.069490
C	-0.863654	-0.390425	-2.085212
C	-0.552699	-1.180194	-0.979406
C	0.718205	-1.739898	-0.857878
C	1.678155	-1.509832	-1.842156
H	2.111609	-0.541655	-3.711236
H	-0.144840	0.452081	-3.927005
H	-1.849198	0.043607	-2.179454
H	-1.297108	-1.358602	-0.216132
H	0.959341	-2.352338	-0.000363
H	2.663699	-1.943864	-1.747914

r=5.0 Å; *R*=3.0 Å

C	3.333373	-0.613814	1.997034
C	4.187244	-0.756089	0.739939
C	5.657221	-0.957340	1.098177
C	5.835063	-2.162068	2.018316
C	4.981189	-2.019793	3.275415
C	3.511216	-1.818547	2.917177
H	4.068573	0.118616	0.098775
H	6.026861	-0.061838	1.606598
H	6.255743	-1.077951	0.194017
H	6.885904	-2.285245	2.284477
H	5.534765	-3.067823	1.483079
H	5.330662	-1.155998	3.848803
H	5.099872	-2.894498	3.916578
H	3.141575	-2.714047	2.408752
H	2.912683	-1.697932	3.821329
H	3.837773	-1.619883	0.166548
H	3.633668	0.291937	2.532280
H	2.282529	-0.490643	1.730884
C	1.336126	-0.801327	-2.997263
C	0.065221	-0.241623	-3.118791
C	-0.894728	-0.471688	-2.134513
C	-0.583773	-1.261458	-1.028707
C	0.687131	-1.821162	-0.907179
C	1.647081	-1.591096	-1.891456
H	2.080534	-0.622919	-3.760537
H	-0.175914	0.370817	-3.976306
H	-1.880272	-0.037657	-2.228754
H	-1.328182	-1.439866	-0.265433
H	0.928266	-2.433602	-0.049663
H	2.632624	-2.025128	-1.797215

r=5.0 Å; *R*=3.1 Å

C	3.333373	-0.613814	1.997034
C	4.187244	-0.756089	0.739939
C	5.657221	-0.957340	1.098177
C	5.835063	-2.162068	2.018316
C	4.981189	-2.019793	3.275415
C	3.511216	-1.818547	2.917177
H	4.068573	0.118616	0.098775
H	6.026861	-0.061838	1.606598
H	6.255743	-1.077951	0.194017
H	6.885904	-2.285245	2.284477
H	5.534765	-3.067823	1.483079
H	5.330662	-1.155998	3.848803
H	5.099872	-2.894498	3.916578
H	3.141575	-2.714047	2.408752
H	2.912683	-1.697932	3.821329
H	3.837773	-1.619883	0.166548
H	3.633668	0.291937	2.532280
H	2.282529	-0.490643	1.730884
C	1.305052	-0.882591	-3.046563
C	0.034147	-0.322887	-3.168092
C	-0.925802	-0.552952	-2.183814
C	-0.614847	-1.342722	-1.078008
C	0.656057	-1.902426	-0.956480
C	1.616007	-1.672360	-1.940757
H	2.049460	-0.704183	-3.809837
H	-0.206988	0.289553	-4.025607
H	-1.911346	-0.118921	-2.278055
H	-1.359256	-1.521130	-0.314734
H	0.897192	-2.514866	-0.098964
H	2.601550	-2.106392	-1.846516

r=5.0 Å; *R*=3.2 Å

C	3.333373	-0.613814	1.997034
C	4.187244	-0.756089	0.739939
C	5.657221	-0.957340	1.098177
C	5.835063	-2.162068	2.018316
C	4.981189	-2.019793	3.275415
C	3.511216	-1.818547	2.917177
H	4.068573	0.118616	0.098775
H	6.026861	-0.061838	1.606598
H	6.255743	-1.077951	0.194017
H	6.885904	-2.285245	2.284477
H	5.534765	-3.067823	1.483079
H	5.330662	-1.155998	3.848803
H	5.099872	-2.894498	3.916578
H	3.141575	-2.714047	2.408752
H	2.912683	-1.697932	3.821329
H	3.837773	-1.619883	0.166548
H	3.633668	0.291937	2.532280
H	2.282529	-0.490643	1.730884
C	1.273978	-0.963855	-3.095864
C	0.003073	-0.404151	-3.217392
C	-0.956877	-0.634216	-2.233115
C	-0.645922	-1.423986	-1.127309
C	0.624983	-1.983690	-1.005780
C	1.584933	-1.753624	-1.990058
H	2.018386	-0.785447	-3.859138
H	-0.238062	0.208289	-4.074908
H	-1.942420	-0.200185	-2.327356
H	-1.390330	-1.602394	-0.364035
H	0.866118	-2.596130	-0.148265
H	2.570476	-2.187656	-1.895817

OH/O

R=1.8 Å

O	1.507513	1.039614	0.000000
H	0.972090	0.243850	0.000000
H	2.406238	0.704633	0.000000
H	0.716592	2.656538	0.000000
O	0.295207	3.517999	0.000000
H	0.196908	3.718957	0.932542

R=1.9 Å

O	1.507513	1.039614	0.000000
H	0.972090	0.243850	0.000000
H	2.406238	0.704633	0.000000
H	0.672652	2.746367	0.000000
O	0.251267	3.607828	0.000000
H	0.152968	3.808786	0.932542

R=2.0 Å

O	1.507513	1.039614	0.000000
H	0.972090	0.243850	0.000000
H	2.406238	0.704633	0.000000
H	0.628712	2.836196	0.000000
O	0.207327	3.697657	0.000000
H	0.109028	3.898615	0.932542

R=2.1 Å

O	1.507513	1.039614	0.000000
H	0.972090	0.243850	0.000000
H	2.406238	0.704633	0.000000
H	0.584772	2.926025	0.000000
O	0.163387	3.787486	0.000000
H	0.065088	3.988444	0.932542

R=2.2 Å

O	1.507513	1.039614	0.000000
H	0.972090	0.243850	0.000000
H	2.406238	0.704633	0.000000
H	0.540832	3.015854	0.000000
O	0.119447	3.877316	0.000000
H	0.021148	4.078274	0.932542

CH/O

R=2.5 Å

C	0.011025	0.000016	0.001899
H	0.004650	-0.000022	1.087301
H	1.036978	-0.000003	-0.352549
H	-0.498009	0.886233	-0.363206
H	-0.498000	-0.886223	-0.363178
O	-0.010032	-0.000110	3.587258
H	0.817656	-0.000153	4.071627
H	-0.843352	-0.000101	4.061872

R=2.6 Å

C	0.011025	0.000016	0.001899
H	0.004650	-0.000022	1.087301
H	1.036978	-0.000003	-0.352549
H	-0.498009	0.886233	-0.363206
H	-0.498000	-0.886223	-0.363178
O	-0.010619	-0.000114	3.687256
H	0.817069	-0.000156	4.171625
H	-0.843939	-0.000105	4.161870

R=2.7 Å

C	0.011025	0.000016	0.001899
H	0.004650	-0.000022	1.087301
H	1.036978	-0.000003	-0.352549
H	-0.498009	0.886233	-0.363206
H	-0.498000	-0.886223	-0.363178
O	-0.011206	-0.000117	3.787254
H	0.816482	-0.000160	4.271624
H	-0.844526	-0.000108	4.261869

R=2.8 Å

C	0.011025	0.000016	0.001899
H	0.004650	-0.000022	1.087301
H	1.036978	-0.000003	-0.352549
H	-0.498009	0.886233	-0.363206
H	-0.498000	-0.886223	-0.363178
O	-0.011794	-0.000121	3.887253
H	0.815894	-0.000163	4.371622
H	-0.845114	-0.000112	4.361867

R=2.9 Å

C	0.011025	0.000016	0.001899
H	0.004650	-0.000022	1.087301
H	1.036978	-0.000003	-0.352549
H	-0.498009	0.886233	-0.363206
H	-0.498000	-0.886223	-0.363178
O	-0.012381	-0.000124	3.987251
H	0.815307	-0.000167	4.471620
H	-0.845701	-0.000115	4.461865

OH/π

R=2.2 Å

C	-1.179992	1.036647	0.000000
C	0.036324	0.356353	0.000000
C	0.055330	-1.037154	0.000000
C	-2.377302	0.323433	0.000000
C	-2.358296	-1.070074	0.000000
C	-1.141980	-1.750368	0.000000
H	-1.194737	2.117677	0.000000
H	0.965151	0.909637	0.000000
H	0.998901	-1.564900	0.000000
H	-3.320873	0.851179	0.000000
H	-3.287123	-1.623358	0.000000
H	-1.127235	-2.831398	0.000000
H	-1.160674	-0.356675	2.200000
O	-1.160674	-0.356675	3.159000
H	-1.961845	-0.833916	3.382711

R=2.3 Å

C	-1.179992	1.036647	0.000000
C	0.036324	0.356353	0.000000
C	0.055330	-1.037154	0.000000
C	-2.377302	0.323433	0.000000
C	-2.358296	-1.070074	0.000000
C	-1.141980	-1.750368	0.000000
H	-1.194737	2.117677	0.000000
H	0.965151	0.909637	0.000000
H	0.998901	-1.564900	0.000000
H	-3.320873	0.851179	0.000000
H	-3.287123	-1.623358	0.000000
H	-1.127235	-2.831398	0.000000
H	-1.160674	-0.356675	2.300000
O	-1.160674	-0.356675	3.259000
H	-1.961845	-0.833916	3.482711

R=2.4 Å

C	-1.179992	1.036647	0.000000
C	0.036324	0.356353	0.000000
C	0.055330	-1.037154	0.000000
C	-2.377302	0.323433	0.000000
C	-2.358296	-1.070074	0.000000
C	-1.141980	-1.750368	0.000000
H	-1.194737	2.117677	0.000000
H	0.965151	0.909637	0.000000
H	0.998901	-1.564900	0.000000
H	-3.320873	0.851179	0.000000
H	-3.287123	-1.623358	0.000000
H	-1.127235	-2.831398	0.000000
H	-1.160674	-0.356675	2.400000
O	-1.160674	-0.356675	3.359000
H	-1.961845	-0.833916	3.582711

R=2.5 Å

C	-1.179992	1.036647	0.000000
C	0.036324	0.356353	0.000000
C	0.055330	-1.037154	0.000000
C	-2.377302	0.323433	0.000000
C	-2.358296	-1.070074	0.000000
C	-1.141980	-1.750368	0.000000
H	-1.194737	2.117677	0.000000
H	0.965151	0.909637	0.000000
H	0.998901	-1.564900	0.000000
H	-3.320873	0.851179	0.000000
H	-3.287123	-1.623358	0.000000
H	-1.127235	-2.831398	0.000000
H	-1.160674	-0.356675	2.500000
O	-1.160674	-0.356675	3.459000
H	-1.961845	-0.833916	3.682711

R=2.6 Å

C	-1.179992	1.036647	0.000000
C	0.036324	0.356353	0.000000
C	0.055330	-1.037154	0.000000
C	-2.377302	0.323433	0.000000
C	-2.358296	-1.070074	0.000000
C	-1.141980	-1.750368	0.000000
H	-1.194737	2.117677	0.000000
H	0.965151	0.909637	0.000000
H	0.998901	-1.564900	0.000000
H	-3.320873	0.851179	0.000000
H	-3.287123	-1.623358	0.000000
H	-1.127235	-2.831398	0.000000
H	-1.160674	-0.356675	2.600000
O	-1.160674	-0.356675	3.559000
H	-1.961845	-0.833916	3.782711

Antiparallel OH/OH

R=1.15 Å

O	0.000000	0.000000	0.000000
H	0.000000	0.000000	0.958000
H	0.927903	0.000000	-0.238245
O	1.399562	-1.149854	-2.020219
H	0.471659	-1.149854	-1.781974
H	1.399562	-1.149854	-2.978219

R=1.25 Å

O	0.000000	0.000000	0.000000
H	0.000000	0.000000	0.958000
H	0.927903	0.000000	-0.238245
O	1.399562	-1.249854	-2.020219
H	0.471659	-1.249854	-1.781974
H	1.399562	-1.249854	-2.978219

R=1.35 Å

O	0.000000	0.000000	0.000000
H	0.000000	0.000000	0.958000
H	0.927903	0.000000	-0.238245
O	1.399562	-1.349854	-2.020219
H	0.471659	-1.349854	-1.781974
H	1.399562	-1.349854	-2.978219

R=1.45 Å

O	0.000000	0.000000	0.000000
H	0.000000	0.000000	0.958000
H	0.927903	0.000000	-0.238245
O	1.399562	-1.449854	-2.020219
H	0.471659	-1.449854	-1.781974
H	1.399562	-1.449854	-2.978219

R=1.55 Å

O	0.000000	0.000000	0.000000
H	0.000000	0.000000	0.958000
H	0.927903	0.000000	-0.238245
O	1.399562	-1.549854	-2.020219
H	0.471659	-1.549854	-1.781974
H	1.399562	-1.549854	-2.978219

Optimized systems—Figs.SIII-1–SIII-8.

Benzene/benzene

C	-1.178289	1.033425	0.038828	C	-1.239022	1.027185	0.008040
C	0.034084	0.353800	0.034542	C	-0.020817	0.359360	0.057545
C	0.051575	-1.035918	0.044717	C	0.010691	-1.028816	0.049205
C	-2.372801	0.323372	0.054049	C	-2.426038	0.306555	-0.046792
C	-2.355196	-1.066158	0.064471	C	-2.394473	-1.084038	-0.054913
C	-1.143043	-1.745772	0.060123	C	-1.175951	-1.750642	-0.008430
H	-1.192108	2.115912	0.038308	H	-1.263166	2.109470	0.021464
H	0.964737	0.906919	0.031001	H	0.902175	0.920207	0.119695
H	0.995994	-1.565105	0.049792	H	0.958305	-1.547887	0.103533
H	-3.317001	0.852623	0.065879	H	-3.374811	0.826961	-0.082549
H	-3.285536	-1.619096	0.084687	H	-3.318644	-1.646544	-0.096893
H	-1.129101	-2.828065	0.076319	H	-1.150914	-2.832985	-0.008128
C	-1.181813	1.031832	3.859814	C	0.381065	1.057443	3.501646
C	0.031839	0.354589	3.863734	C	1.586699	0.367352	3.540834
C	0.052050	-1.035093	3.855297	C	1.591061	-1.023620	3.539722
C	-2.374958	0.319491	3.846133	C	0.389753	-1.721167	3.498676
C	-2.354708	-1.070023	3.837355	C	-0.819900	0.358660	3.457769
C	-1.141219	-1.747318	3.841671	C	-0.815529	-1.029910	3.455673
H	-1.197685	2.114258	3.858419	H	0.377116	2.140071	3.496716
H	0.961410	0.909484	3.864853	H	2.522032	0.911850	3.572591
H	0.997499	-1.562471	3.850038	H	2.529805	-1.562221	3.570905
H	-3.320172	0.846900	3.834278	H	0.392668	-2.803761	3.491175
H	-3.284007	-1.624782	3.818345	H	-1.757603	0.895899	3.408252
H	-1.125184	-2.829634	3.826743	H	-1.749780	-1.572946	3.404265

Benzene/pyridine

C	-0.986479	0.702421	0.407955
C	0.041739	-0.213599	0.251252
C	-0.275306	-1.507272	-0.139713
C	-2.289428	0.282912	0.170057
N	-2.608621	-0.955829	-0.202824
C	-1.610167	-1.827310	-0.351864
H	-0.790661	1.716504	0.727449
H	1.068056	0.071182	0.441912
H	0.492611	-2.257999	-0.271508
H	-3.116546	0.973898	0.293349
H	-1.891856	-2.830534	-0.654676
C	-2.053651	-0.977450	3.371976
C	-2.367784	0.340184	3.684864
C	-1.363561	1.225425	4.058641
C	-0.043216	0.792622	4.120286
C	0.270172	-0.525886	3.811457
C	-0.735400	-1.410186	3.437087
H	-2.833232	-1.659840	3.060727
H	-3.394698	0.677841	3.628830
H	-1.608512	2.251944	4.300679
H	0.739445	1.481845	4.411451
H	1.297895	-0.863233	3.858351
H	-0.490234	-2.433329	3.184263

Pyridine/pyridine

C	-1.499933	1.827753	-0.333210
C	-0.537907	1.065103	0.312493
C	-0.864729	-0.227025	0.699600
C	-2.748185	1.263527	-0.562554
N	-3.076399	0.025072	-0.193841
C	-2.143255	-0.697933	0.425348
H	-1.296534	2.845107	-0.637172
H	0.442605	1.474276	0.518389
H	-0.148324	-0.859358	1.207222
H	-3.523932	1.837122	-1.057732
H	-2.431317	-1.702540	0.717526
N	-1.978546	3.914892	2.518382
C	-3.095916	4.327582	1.919277
C	-4.233978	3.540308	1.792920
C	-4.212615	2.254968	2.316732
C	-3.058682	1.816531	2.948386
C	-1.973266	2.680399	3.020761
H	-3.079943	5.335755	1.518242
H	-5.110724	3.926137	1.289744
H	-5.070487	1.602904	2.220079
H	-2.989967	0.819266	3.360466
H	-1.053010	2.362184	3.498943

Benzene/cyclohexane

C	3.319528	-0.627846	1.978091	C	3.231681	-0.628739	2.174347
C	4.184564	-0.763023	0.723393	C	3.901456	-0.777078	0.806829
C	5.656171	-0.984354	1.080068	C	5.406879	-1.014658	0.940717
C	5.829666	-2.186987	2.009834	C	5.706891	-2.210951	1.846202
C	4.965044	-2.053482	3.265015	C	5.038834	-2.058498	3.214550
C	3.493946	-1.831002	2.907016	C	3.532447	-1.826796	3.077059
H	4.076834	0.123376	0.092380	H	3.713123	0.110559	0.196918
H	6.046623	-0.086711	1.573832	H	5.878809	-0.119176	1.361858
H	6.247426	-1.122421	0.170865	H	5.851666	-1.168355	-0.046008
H	6.881362	-2.303878	2.285109	H	6.786641	-2.335710	1.963922
H	5.539110	-3.095158	1.473481	H	5.333628	-3.121274	1.366585
H	5.323599	-1.206653	3.862004	H	5.487122	-1.207904	3.741280
H	5.070389	-2.943491	3.891220	H	5.231180	-2.941717	3.829641
H	3.111163	-2.725357	2.406160	H	3.071707	-2.720986	2.645309
H	2.899795	-1.697440	3.815157	H	3.080419	-1.682432	4.061994
H	3.828664	-1.615371	0.136764	H	3.451355	-1.620206	0.277547
H	3.601740	0.285934	2.514184	H	3.595125	0.285161	2.658794
H	2.267950	-0.515043	1.700956	H	2.151786	-0.510243	2.052747
C	2.340497	-5.006101	0.942325	C	3.507382	-3.767211	-1.734303
C	3.610902	-5.558868	1.058603	C	2.324546	-3.060435	-1.920478
C	4.562969	-5.325482	0.072778	C	1.315925	-3.129024	-0.965772
C	4.245118	-4.538848	-1.028515	C	1.490156	-3.904628	0.174851
C	2.974776	-3.986118	-1.144638	C	2.672146	-4.612626	0.359882
C	2.022395	-4.219315	-0.158990	C	3.680525	-4.544125	-0.594538
H	1.600325	-5.184423	1.711888	H	4.294624	-3.709647	-2.474989
H	3.860009	-6.167101	1.918648	H	2.191268	-2.451854	-2.805680
H	5.553311	-5.752407	0.164978	H	0.397337	-2.574377	-1.108077
H	4.987901	-4.353078	-1.793540	H	0.707745	-3.953256	0.921197
H	2.728176	-3.370668	-2.000212	H	2.809913	-5.212539	1.250180
H	1.034735	-3.784883	-0.246445	H	4.602462	-5.091948	-0.448354

C	3.183869	-0.393356	1.123691
C	4.489824	-0.671735	0.378230
C	5.634167	-0.980083	1.345510
C	5.272978	-2.131609	2.286855
C	3.966727	-1.854106	3.034464
C	2.825006	-1.544365	2.064529
H	4.748817	0.176586	-0.260712
H	5.852197	-0.086370	1.941644
H	6.547019	-1.216110	0.792149
H	6.085382	-2.310463	2.996087
H	5.161565	-3.051448	1.700882
H	4.112829	-0.997971	3.703276
H	3.706368	-2.705020	3.669589
H	2.609066	-2.435557	1.465574
H	1.909604	-1.311667	2.614926
H	4.340842	-1.528113	-0.287970
H	3.294019	0.527389	1.709015
H	2.374332	-0.221495	0.414082
C	1.613273	-0.420266	-2.454093
C	0.503394	0.090685	-1.790724
C	-0.201471	-0.708473	-0.897073
C	0.203415	-2.018462	-0.667572
C	1.312404	-2.529514	-1.332101
C	2.017230	-1.730694	-2.224924
H	2.165470	0.203216	-3.145429
H	0.190738	1.112007	-1.966066
H	-1.062810	-0.309177	-0.377079
H	-0.341076	-2.638611	0.032718
H	1.630415	-3.548036	-1.149888
H	2.884434	-2.126846	-2.737227

OH/O

O	1.487958	0.948972	0.304625
H	0.928714	0.303188	-0.141186
H	2.324937	0.943580	-0.172846
H	0.683846	2.697850	0.158557
O	0.285003	3.561043	-0.036606
H	0.120450	3.965932	0.819998

CH/O

C	0.011626	-0.000006	0.041507
H	0.010471	-0.000040	1.130570
H	1.038028	0.000006	-0.325334
H	-0.500661	0.888809	-0.326553
H	-0.500661	-0.888797	-0.326610
O	-0.010448	-0.000108	3.657210
H	0.750615	-0.000148	4.246732
H	-0.781577	-0.000100	4.233493

OH/π

C	-1.223654	1.007810	0.063804
C	-0.009695	0.328140	0.076410
C	0.009985	-1.062963	0.061522
C	-2.417949	0.296316	0.034493
C	-2.398181	-1.094723	0.017972
C	-1.184309	-1.774386	0.032240
H	-1.238595	2.089834	0.079862
H	0.920247	0.882137	0.096839
H	0.954405	-1.591277	0.075577
H	-3.362453	0.824554	0.027776
H	-3.327973	-1.648623	-0.007156
H	-1.169491	-2.856447	0.023714
H	-0.602968	-0.021720	2.735994
O	-1.263137	-0.414147	3.318452
H	-1.901257	-0.794096	2.704212

Antiparallel OH/OH

O	-0.684514	0.758213	0.967986
H	-0.686580	0.563709	1.910632
H	0.237755	0.690364	0.687108
O	0.684682	-0.758079	-0.968162
H	-0.237475	-0.690788	-0.686790
H	0.686131	-0.563420	-1.910774

SI-III. Optimizations of the obtained minima

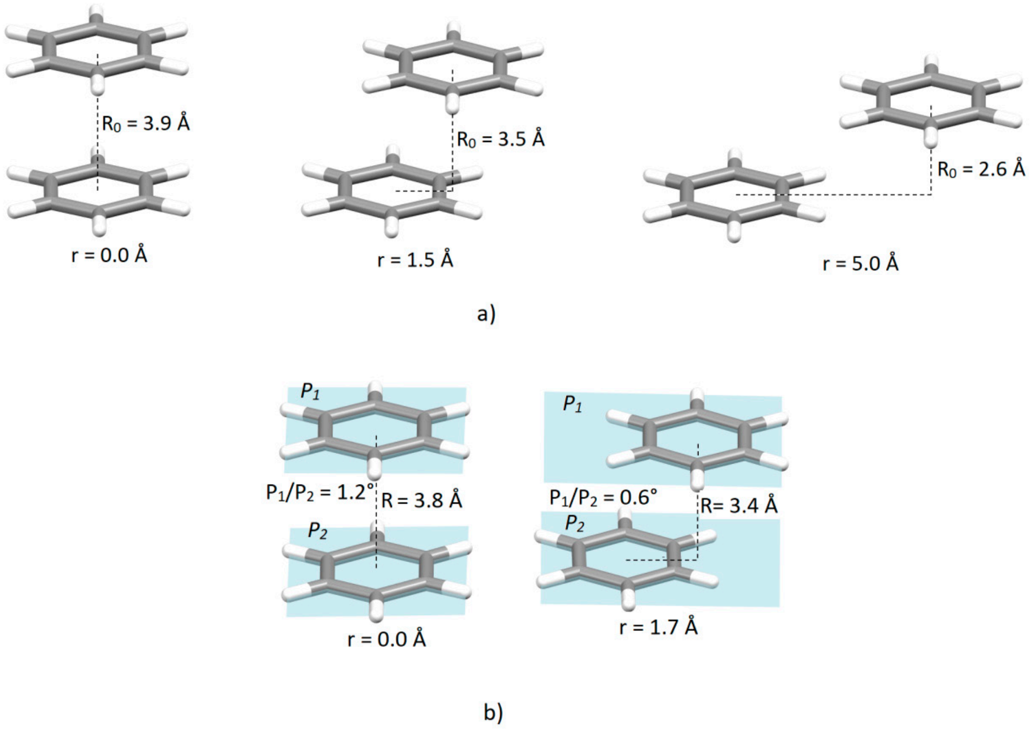

Figure SIII-1: a) Starting geometries obtained as the potential curve minima of the benzene/benzene system and b) the corresponding optimized geometries starting from r=0.0 Å (left) and r=1.5 Å and r=5.0 Å (right)

Table SIII-1: Total energies and the SAPT decomposition of the benzene/benzene interaction energies (kcal/mol) of the minimum and optimized geometries

	r=0.0 Å, minimum	r=0.0 Å, optimized	r=1.5 Å, minimum	r=5.0 Å, minimum	r=1.5 Å, r=5.0 Å, optimized
E total	-291609.17	-291609.33	-291610.32	-291609.23	-291610.53
ELST.	0.10	-0.08	-1.51	-1.36	-1.90
EXCH-REP.	3.30	4.27	6.63	2.85	7.23
IND.	-0.22	-0.27	-0.71	-0.31	-0.80
DISP.	-5.01	-5.16	-7.22	-3.14	-6.78
NET DISP.	-1.71	-0.90	-0.59	-0.30	0.44
E int	-1.83	-1.25	-2.81	-1.96	-2.26

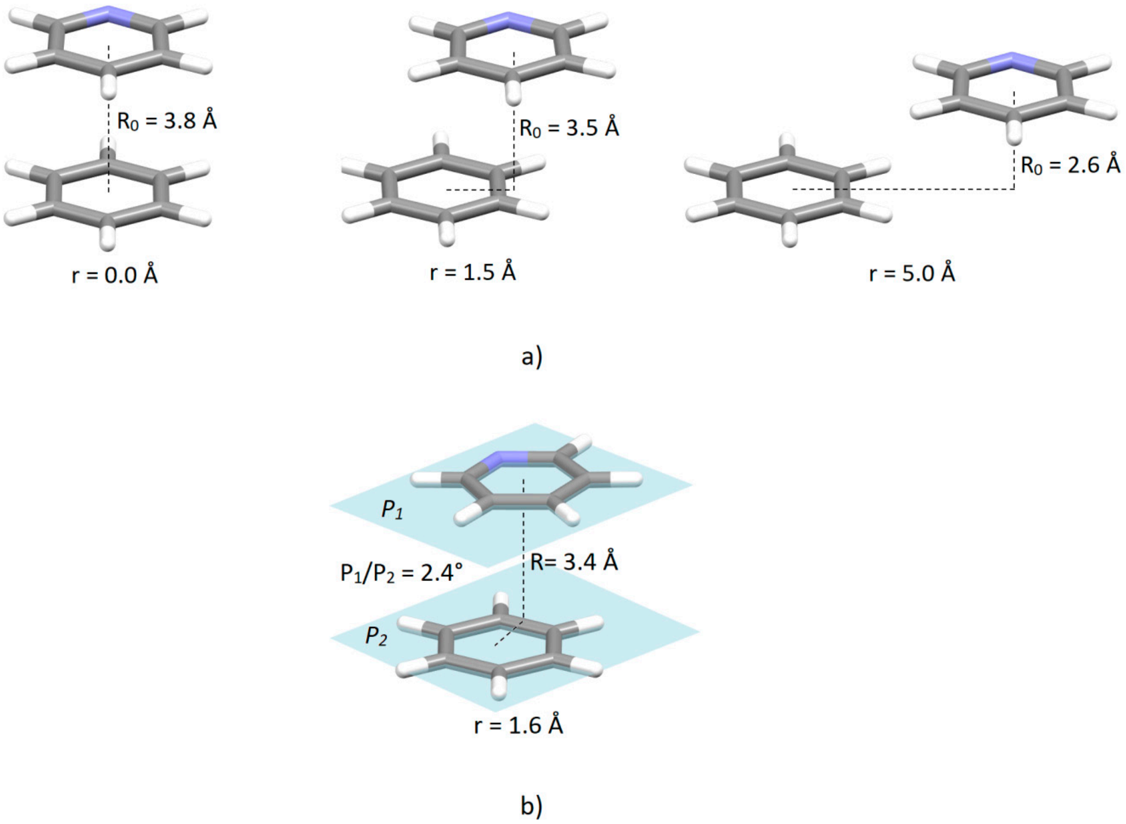

Figure SIII-2: a) Starting geometries obtained as the potential curve minima of the benzene/pyridine system and b) the corresponding optimized geometry, which is the same for all systems

Table SIII-2: Total energies and the SAPT decomposition of the benzene/pyridine interaction energies (kcal/mol) of the minimum and optimized geometries

	r=0.0 Å, minimum	r=1.5 Å, minimum	r=5.0 Å, minimum	r=0.0 Å, r=1.5 Å, r=5.0 Å, optimized
E total	-301672.58	-301675.71	-301674.20	-301676.20
ELST.	-0.52	-1.73	-1.30	-2.56
EXCH-REP.	3.89	5.87	2.55	7.41
IND.	-0.25	-0.64	-0.28	-0.81
DISP.	-5.46	-6.84	-2.95	-6.99
NET DISP.	-1.57	-0.97	-0.41	0.42
E int	-2.33	-3.35	-1.99	-2.95

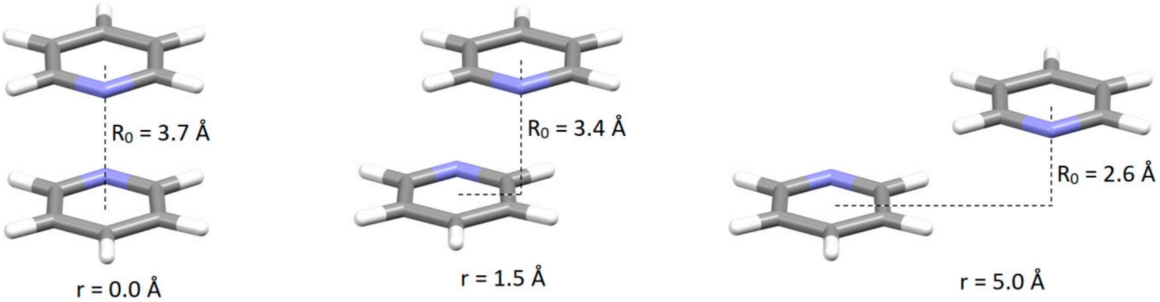

a)

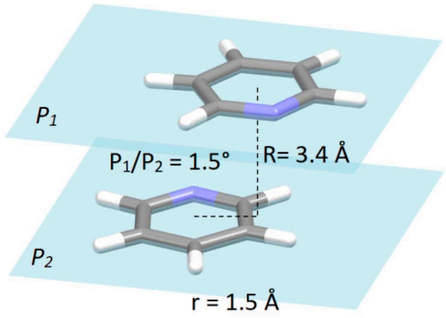

b)

Figure SIII-3: Starting geometries obtained as the potential curve minima of the pyridine/pyridine system and b) the corresponding optimized geometry, which is the same for all systems

Table SIII-3: Total energies and the SAPT decomposition of the pyridine/pyridine interaction energies (kcal/mol) of the minimum and optimized geometries

	r=0.0 Å, minimum	r=1.5 Å, minimum	r=5.0 Å, minimum	r=0.0 Å, r=1.5 Å, r=5.0 Å, optimized
E total	-311738.61	-311739.50	-311737.44	-311741.76
ELST.	-1.39	-2.82	-1.34	-3.14
EXCH-REP.	4.53	6.86	2.23	7.39
IND.	-0.30	-0.73	-0.26	-0.78
DISP.	-5.95	-7.47	-2.76	-7.09
NET DISP.	-1.42	-0.61	-0.53	0.30
E int	-3.11	-4.16	-2.12	-3.62

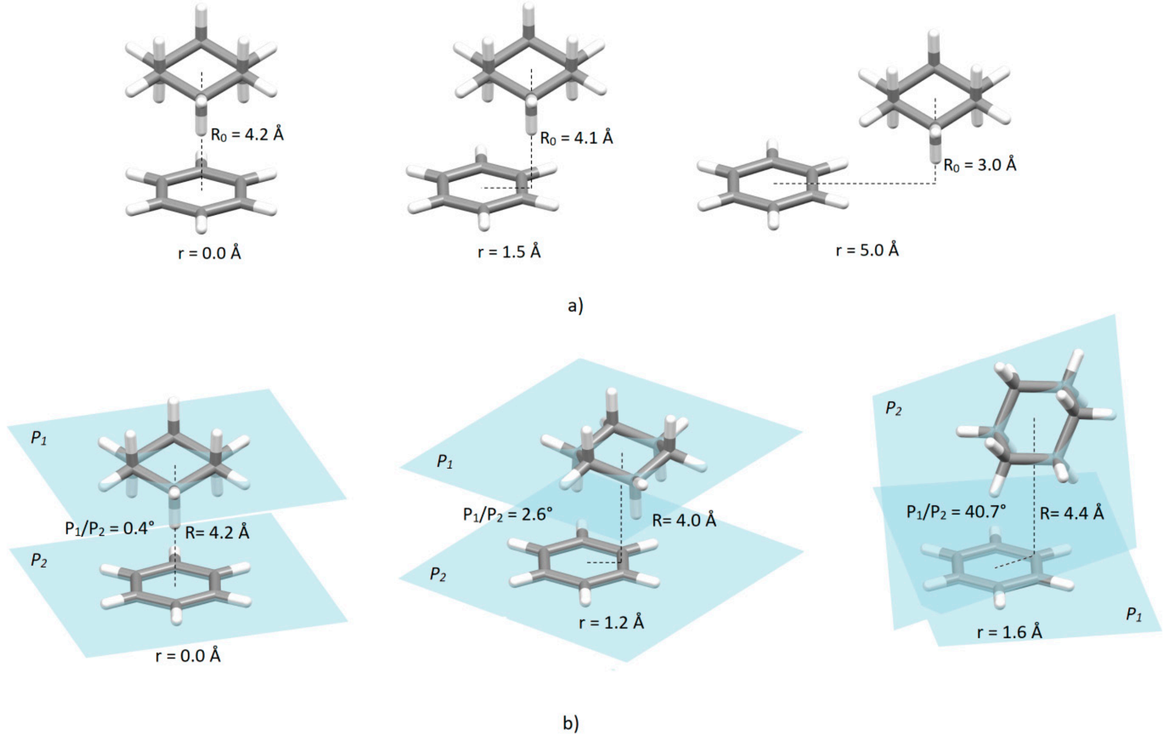

Figure SIII-4: Starting geometries obtained as the potential curve minima of the benzene/cyclohexane system and b) the corresponding optimized geometries, starting from r=0.0 Å (left), r=1.5 Å (middle) and r=5.0 Å (right)

Table SIII-4: Total energies and the SAPT decomposition of the benzene/cyclohexane interaction energies (kcal/mol) of the minimum and optimized geometries

	r=0.0 Å, minimum	r=0.0 Å, optimized	r=1.5 Å, minimum	r=1.5 Å, optimized	r=5.0 Å, minimum	r=5.0 Å, optimized
E total	-293892.79	-293893.01	-293892.45	-293893.07	-293890.78	-293892.79
ELST.	-1.87	-2.21	-1.60	-2.12	-0.53	-1.74
EXCH-REP.	4.99	5.84	4.49	5.74	2.04	4.88
IND.	-0.63	-0.73	-0.52	-0.64	-0.20	-0.52
DISP.	-5.68	-5.53	-5.33	-5.80	-2.72	-5.19
NET DISP.	-0.69	0.31	-0.84	-0.06	-0.68	-0.31
E int	-3.19	-2.63	-2.97	-2.82	-1.41	-2.57

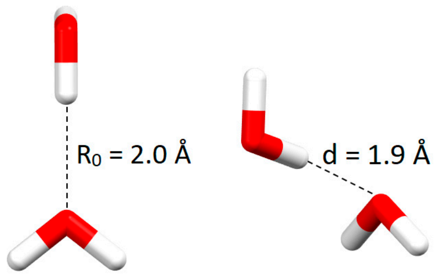

Figure SIII-5: Starting geometry obtained as the potential curve minima (left) and the corresponding optimized geometry (right) of the water/water OH/O interaction

Table SIII-5: Total energies and the SAPT decomposition of the OH/O interaction energies (kcal/mol) of the minimum and optimized geometries

	minimum	optimized
E total	-95967.07	-95968.01
ELST.	-6.39	-9.20
EXCH-REP.	5.79	8.77
IND.	-1.83	-2.55
DISP.	-1.97	-1.89
NET DISP.	3.82	6.89
E int	-4.39	-4.86

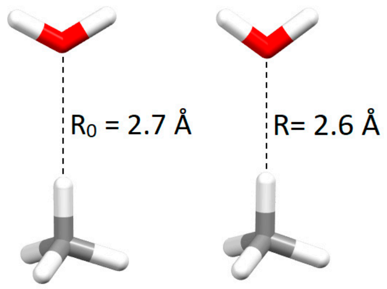

Figure SIII-6: Starting geometry obtained as the potential curve minima (left) and the corresponding optimized geometry (right) of the methane/water CH/O interaction

Table SIII-6: Total energies and the SAPT decomposition of the CH/O interaction energies (kcal/mol) of the minimum and optimized geometries

	minimum	optimized
E total	-73417.27	-73420.19
ELST.	-0.64	-1.00
EXCH-REP.	0.93	1.52
IND.	-0.24	-0.39
DISP.	-0.67	-0.62
NET DISP.	0.26	0.89
E int	-0.63	-0.49

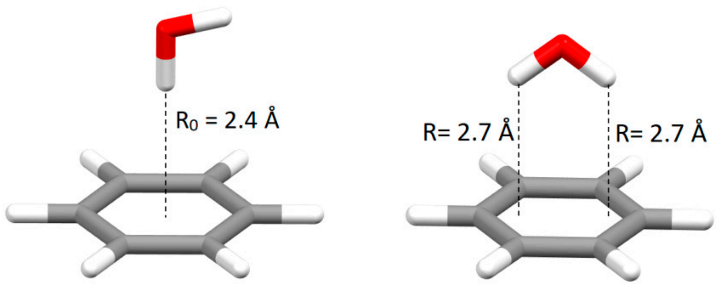

Figure SIII-7: Starting geometry obtained as the potential curve minima (left) and the corresponding optimized geometry (right) of the benzene/water OH/π interaction

Table SIII-7: Total energies and the SAPT decomposition of the OH/π interaction energies (kcal/mol) of the minimum and optimized geometries

	minimum	optimized
E total	-193788.35	-193789.09
ELST.	-2.50	-3.49
EXCH-REP.	3.43	4.70
IND.	-0.97	-0.99
DISP.	-3.07	-2.99
NET DISP.	0.36	1.72
E int	-3.11	-2.77

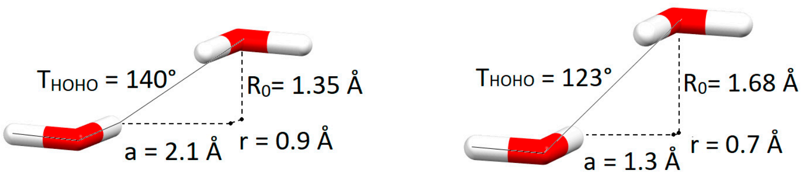

Figure SIII-8: Starting geometry obtained as the potential curve minima (left) and the corresponding optimized geometry (right) of the water/water antiparallel OH/OH interaction

Table SIII-8: Total energies and the SAPT decomposition of the antiparallel OH/OH interaction energies (kcal/mol) of the minimum and optimized geometries

	minimum	optimized
E total	-95966.93	-95967.31
ELST.	-6.36	-8.13
EXCH-REP.	5.63	7.35
IND.	-1.12	-1.39
DISP.	-2.10	-1.74
NET DISP.	3.53	5.61
E int	-3.95	-3.91
